# Supplementary material for: Asynchronous Technical Feedback: A Workshop for Training Surgical Instructors
Source: MedEdPORTAL. 2025 Apr 25;21:11519. doi: 10.15766/mep_2374-8265.11519 (PMC12022122; doi:10.15766/mep_2374-8265.11519)
Supplement: Supplementary file 1 — Facilitator Guide.docxSlides.pptxSmall Group Video 1.mp4Small Group Video 2.mp4Small Group Video 3.mp4Questionnaire.docx [file mep_2374-8265.11519-s001.zip › A. Facilitator Guide.docx]

**Appendix A - Facilitator Guide**

*Use this appendix to plan and run the workshop.*

***Before the Workshop***

Prior to the workshop, decide whether the workshop will be held virtually or in-person. Recruit participants for a one-hour time period. Download Appendices B-F and review materials. If possible, identify the software that your program will use to provide asynchronous technical feedback such that the software can be piloted by participants (with the videos from Appendices C, D, and E) during the workshop.

***During the Workshop***

Slide 1 (5 minutes): Introduce the workshop and the facilitators. Depending on group size, have participants introduce themselves, their experience with feedback, and their motivation for learning more about asynchronous technical feedback.

Slide 2 (1 minute): Review the session objectives. Advise participants that a post-workshop questionnaire will assess the first three objectives and that their participation in group activities during the workshop will assess the fourth objective.

Slide 3 (1 minute): Emphasize that the workshop will start by addressing technical feedback more broadly before honing in on asynchronous technical feedback. Briefly define asynchronous technical feedback.

Slide 4 (1 minute): Discuss the plan for the remainder of the hour and highlight that participants can keep track of workshop progress at the bottom of each slide.

Slide 5 (3 minutes): Ask participants what challenges they have faced providing feedback about technical skills. Note that these challenges do not have to be specific to asynchronous technical feedback. If participants do not offer examples, probe about challenges with technical feedback in operative settings, non-operative clinical settings, and simulation settings.

Slide 6 (3 minutes): Highlight some known challenges associated with providing technical feedback, and discuss how participants’ stated challenges from the prior slide fit into these challenges.

Bullet Point 1: Time is one of the most cited barriers to providing feedback in many settings, including clinically and asynchronously.

Bullet Point 2: Ambiguous feedback and instruction have been shown to be extremely common in procedural and surgical settings. Directional or deictic expressions (e.g., “right,” “left,” “up,” “down,” “here,” “there”) may confuse trainees.

Bullet Point 3: Actionable feedback is one of the most important considerations among trainees. Generically positive or negative feedback does little to advance trainees’ technical skills.

Bullet Point 4: Trainees have more difficulty implementing feedback when the feedback giver is intimidating or unknown. Trainees may not inquire about feedback because they feel summatively assessed.

Bullet Point 5: Feedback is less effective when trainees cannot focus on the feedback. Extraneous cognitive load – including from the other challenges discussed like lack of clarity – can reduce trainees’ understanding of feedback.

Slide 7 (2 minutes): Talk about the many aspects of effective feedback, starting broadly. Discuss published and widely used models for giving feedback, including the ADAPT model and the start-stop-continue model. Note that these models can provide a framework for thinking about the feedback conversation.

Slide 8 (3 minutes): Ask participants if they keep other considerations in mind when giving feedback. If participants do not offer examples, ask them to reflect on prior feedback conversations and what went well or poorly.

Slide 9 (1 minute): Note that you will discuss five important strategies to provide high-quality feedback based on previously published work. These strategies can be remembered using the “REACT” mnemonic. Also discuss that there are many other considerations when providing feedback and that you would welcome participants’ insights from their own experiences after discussing these five strategies.

Slide 10 (1 minute): First, discuss the importance of establishing a relationship and expectations. Trainees know when attendings are invested in their development. Prior work has shown the importance of this, including a qualitative study that included the listed quote. Relationships with mutual respect and investment improve feedback and speed technical skill acquisition.

Slide 11 (1 minute): Second, highlight the importance of incorporating both reinforcing and corrective feedback when providing asynchronous technical feedback. It is crucial for feedback givers to tell trainees what they should keep doing (reinforcing feedback) as well as what they should change (corrective feedback). Prior studies have established the usefulness of reinforcing feedback.

Slides 12-14 (2 minutes): Third, discuss the idea of the zone of proximal development, or the sweet spot in which trainees learn best. Asking a chief resident to place a urinary catheter would likely not further that resident’s skill. Conversely, a first-year medical student with no training might be unable to complete the task, leading only to stress. A third-year medical student who has seen a catheter insertion and practiced on a manikin would be in the perfect ‘zone’ to perform this task. While the zone of proximal development is often thought of with regard to tasks (i.e., what tasks should we have trainees practice to best improve their skill) it can also be a useful way to think about feedback. For example, a trainee really struggling with the basics of a task would likely not benefit from nuanced or high-level feedback.

Slide 15 (2 minutes): Fourth, introduce the concept of multimodality, or the harnessing of all available tools to provide feedback. Purely written feedback can be challenging for trainees to understand. Drawings or audio may supplement written comments to improve clarity and trainee engagement with feedback. Multimodality decreases trainees’ cognitive load and gives them more bandwidth to learn. Provide examples of multimodality, such as accompanying a drawn arrow with an oral description of a way to improve technique. Alternately, one could overlay text on a still frame of a video with a visual depiction of how to move one’s hands or instruments.

Slide 16 (1 minute): Finally, note that feedback should be provided as soon as possible. Trainees benefit from reflection on their performance while the tasks are still fresh in their minds. After discussing this final point, ask if participants have other strategies not listed here to provide high-quality feedback.

Slide 17 (2 minutes): Discuss the role of asynchronous feedback in technical skill development. Busy clinical and simulation settings sometimes preclude appropriate in-person, synchronous feedback. Prior work has shown asynchronous feedback to be similarly effective to synchronous feedback, and that it is particularly suited to technical skill practice and development. Asynchronous feedback can be used for technical skill development for home practice, for practice at a skills center, or for operative video review.

Slide 18 (1 minute): Note that multiple studies have investigated asynchronous technical feedback and have shown it to be efficacious in improving skills.

Slide 19 (2 minutes): Highlight two major barriers to giving asynchronous technical feedback: motivation and technology. These challenges can affect both feedback givers and feedback recipients. To reduce the impact of these barriers, it can be useful (if feasible) to hold a synchronous session with both feedback givers and receivers prior to starting a curriculum. This session can allow those giving and receiving feedback to establish rapport and set expectations for task completion and the timeliness of feedback. A synchronous session also gives participants the opportunity to gain intrinsic motivation to complete the curriculum. Finally, a synchronous session can ensure that both feedback givers and receivers have a chance to practice with the technology that will be used to give and receive feedback. Practicing with the technology before starting a program with asynchronous feedback can reduce strain on program facilitators and decrease user frustration.

Slide 20 (1 minute): Discuss how asynchronous feedback ideally makes use of the same best feedback practices highlighted previously. You can note that some of these best practices will manifest differently. For example, timely asynchronous technical feedback may not be given until some hours later, but should still be given as close to an activity as possible.

Slide 21 (5 minutes): Tell the group that you will be watching a video of a trainee performing a task as part of a home basic open surgical skill curriculum. The trainee was instructed to tie six square knots atraumatically. Watch the video through once, and then ask participants what the trainee did well and where the trainee struggled. Then advise participants that you will watch the video a second time. This time, ask participants to suggest specific time points when they would provide feedback, keeping in mind the best practices previously discussed. After finishing this video, also discuss the usefulness of freeform (narrative) comments and rubric-based scoring to scaffold feedback.

Slides 22-23 (2 minutes): Ask the group how the provided feedback could be improved. The first example (i.e., “You are traumatizing the tissue”) is not actionable and could benefit from the use of multimodality to point out where the trainee is lifting the instrument off the table. The second example (i.e., “Nice job overall”) is generically positive and could benefit from including a specific reinforcing comment.

Slides 24-25 (10 minutes): Split the participants into groups (ideally small groups of two to three participants). Have each group choose one of the three small groups videos to review (Appendices C, D, and E). Depending on the participants, you can also have all small groups review the same video. For example, participants with less technical skill experience may be best equipped to review Appendix C. Have groups repeat the large group practice activity of providing feedback using best practices. The videos are all one to one and a half minutes. Provide groups about ten minutes so that they can watch videos through twice and discuss feedback strategies. During small group sessions, the facilitators should circulate among groups and encourage best practices. Facilitators can also assess participants’ feedback at this time.

Slide 26 (5 minutes): Bring small groups back together and discuss what they gave feedback about and challenges that they encountered while providing feedback. Then talk about how asynchronous feedback differs from in-person, with an emphasis on the different multimodal tools available, changes in the social dynamic, and the ability to review multiple times.

Slide 27 (3 minutes): Ask participants to reflect on what they have learned during the session and respond to any questions that have arisen.

Slide 28 (2 minutes): Summarize the key takeaways from the workshop.

***After the Workshop***

Distribute the questionnaire (Appendix F) to participants to assess their attitudes and knowledge.
