# Supplementary material for: Asynchronous Technical Feedback: A Workshop for Training Surgical Instructors
Source: MedEdPORTAL. 2025 Apr 25;21:11519. doi: 10.15766/mep_2374-8265.11519 (PMC12022122; doi:10.15766/mep_2374-8265.11519)
Supplement: Supplementary file 1 — Facilitator Guide.docxSlides.pptxSmall Group Video 1.mp4Small Group Video 2.mp4Small Group Video 3.mp4Questionnaire.docx [file mep_2374-8265.11519-s001.zip › B. Slides.pptx]

## Slide 1
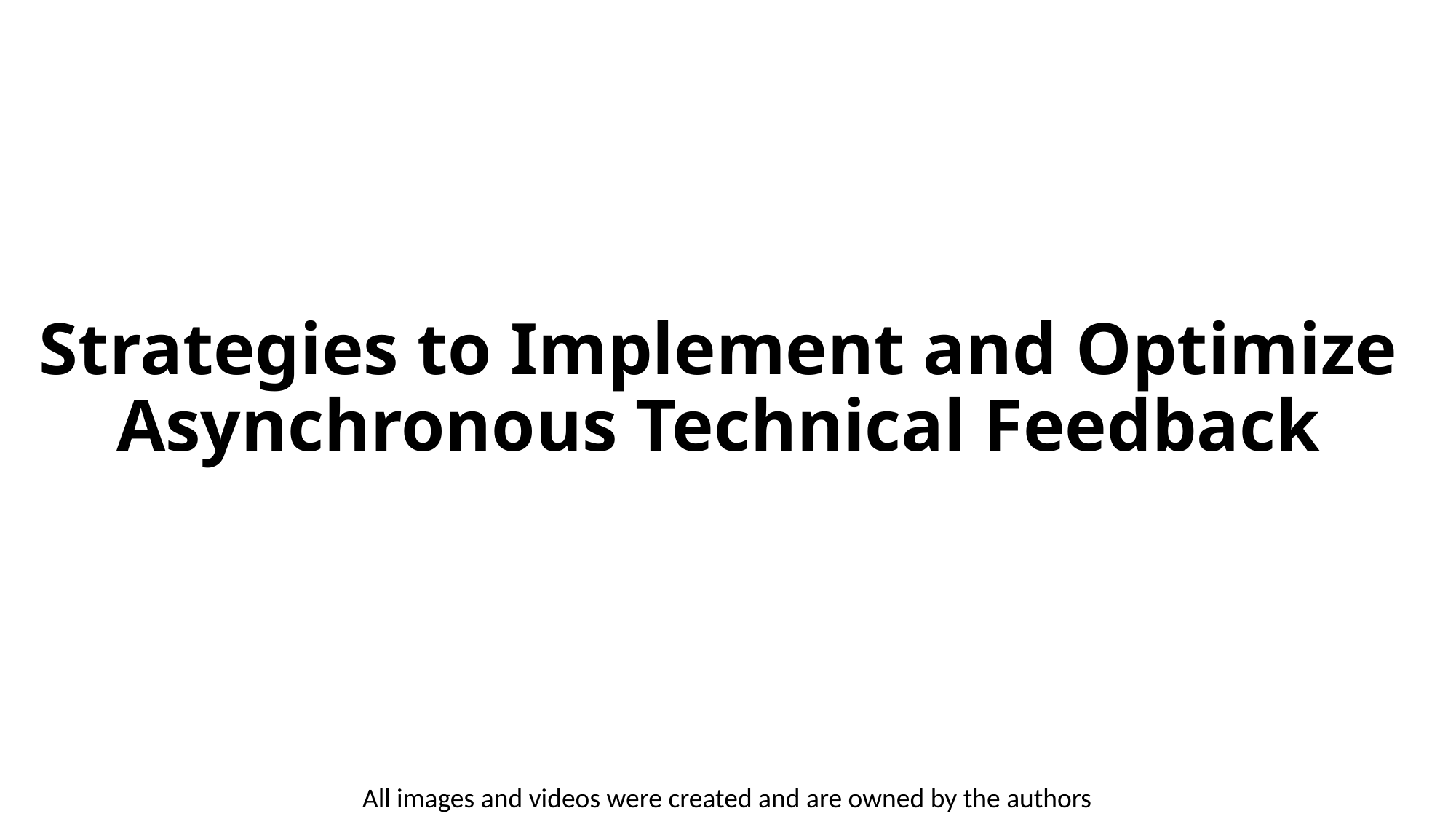

# Strategies to Implement and Optimize Asynchronous Technical Feedback
All images and videos were created and are owned by the authors

## Slide 2
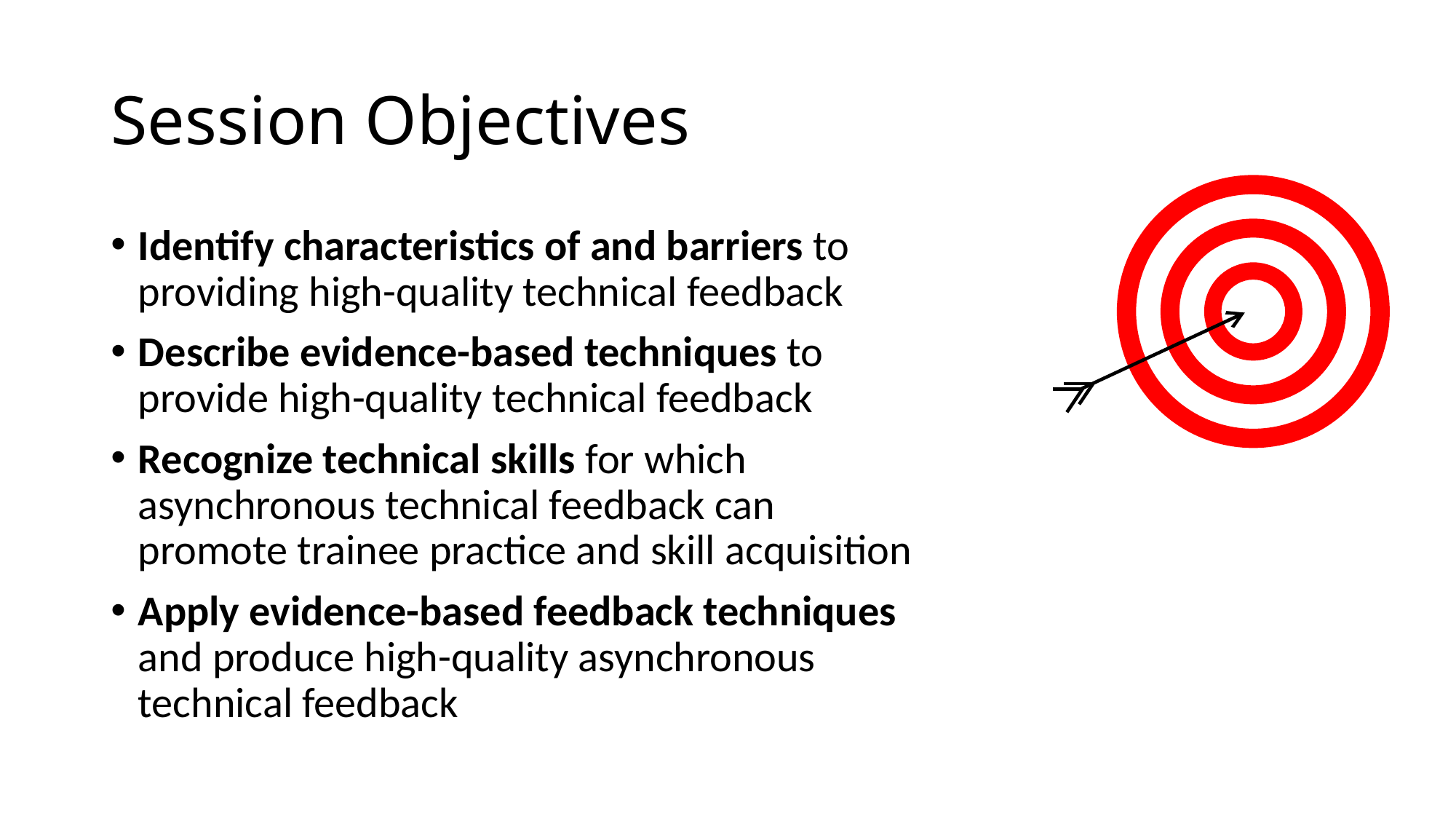

# Session Objectives
Identify characteristics of and barriers to providing high-quality technical feedback
Describe evidence-based techniques to provide high-quality technical feedback
Recognize technical skills for which asynchronous technical feedback can promote trainee practice and skill acquisition
Apply evidence-based feedback techniques and produce high-quality asynchronous technical feedback

## Slide 3
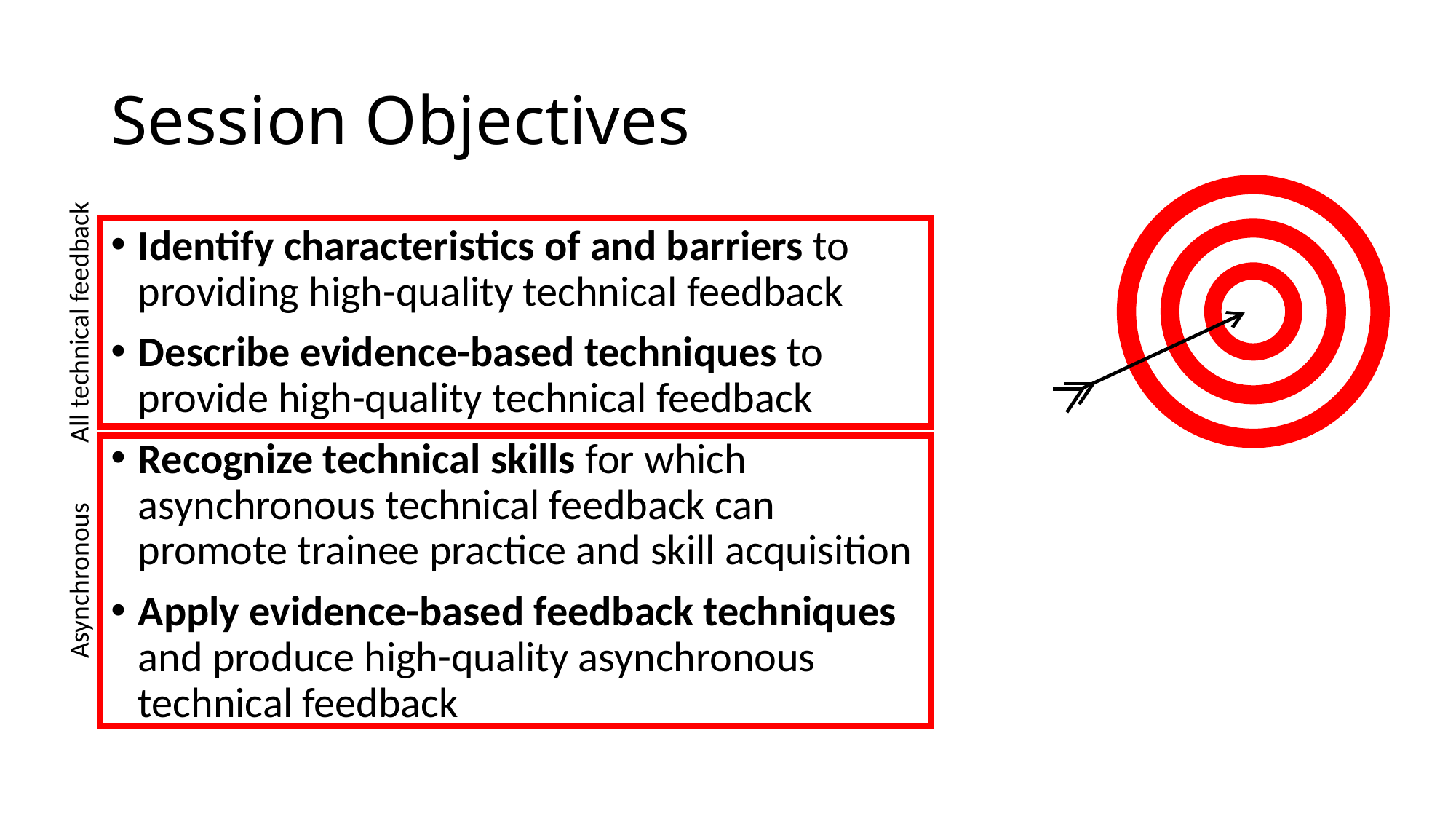

# Session Objectives
Identify characteristics of and barriers to providing high-quality technical feedback
Describe evidence-based techniques to provide high-quality technical feedback
Recognize technical skills for which asynchronous technical feedback can promote trainee practice and skill acquisition
Apply evidence-based feedback techniques and produce high-quality asynchronous technical feedback
All technical feedback
Asynchronous

## Slide 4
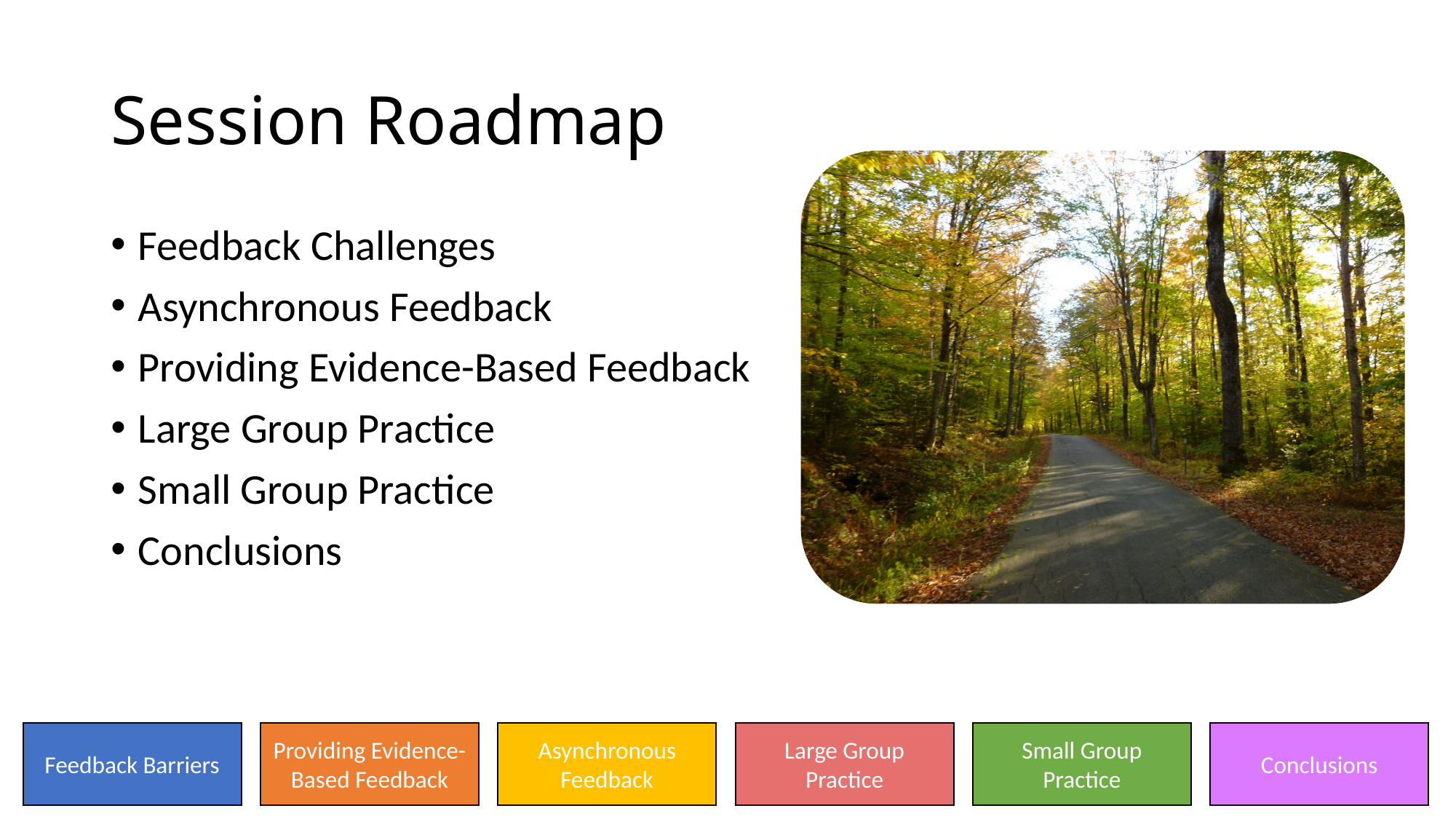

# Session Roadmap
Feedback Challenges
Asynchronous Feedback
Providing Evidence-Based Feedback
Large Group Practice
Small Group Practice
Conclusions
Feedback Barriers
Providing Evidence-Based Feedback
Asynchronous Feedback
Large Group Practice
Small Group Practice
Conclusions

## Slide 5
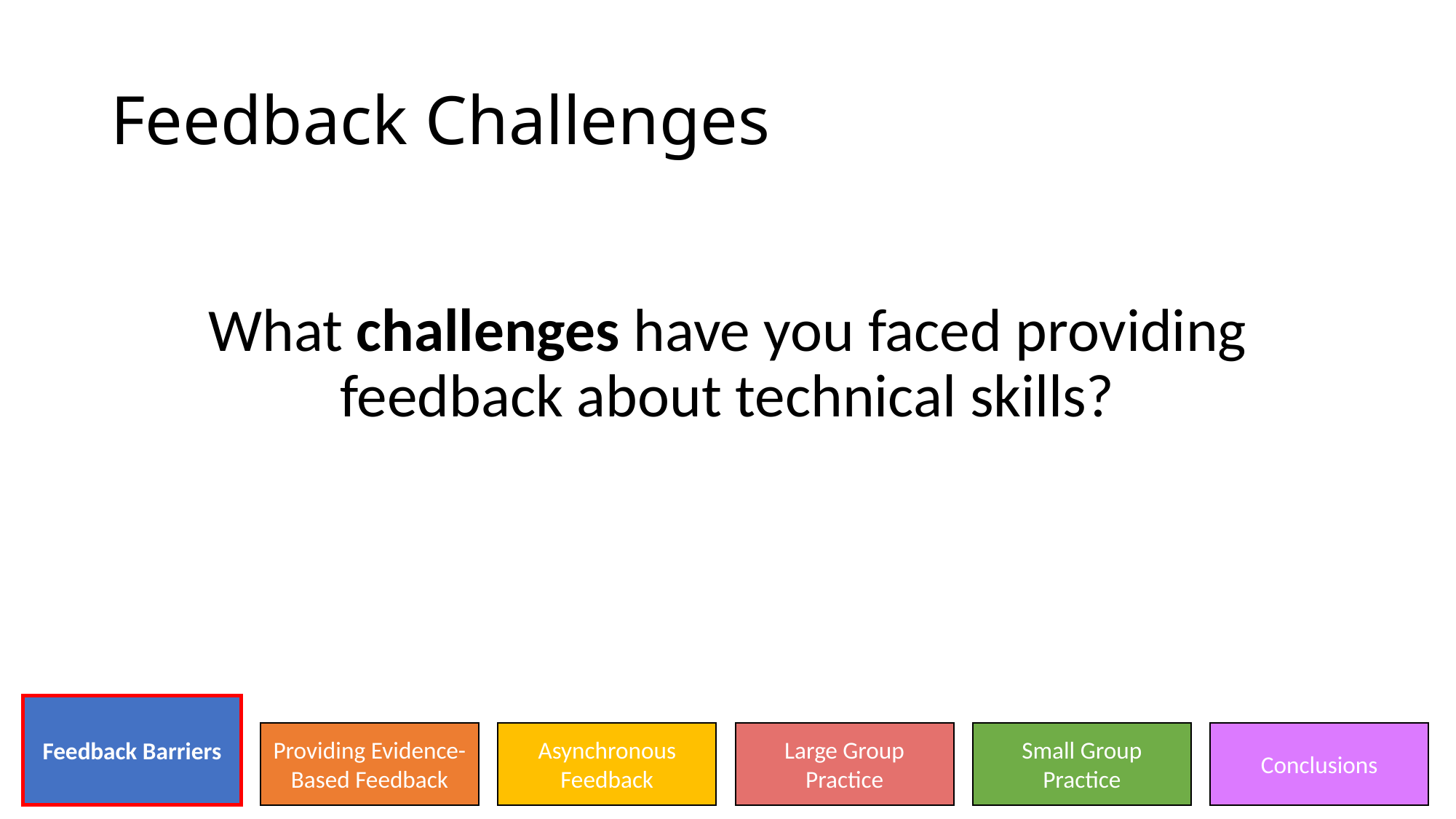

# Feedback Challenges
What challenges have you faced providing feedback about technical skills?
Feedback Barriers
Providing Evidence-Based Feedback
Asynchronous Feedback
Large Group Practice
Small Group Practice
Conclusions

## Slide 6
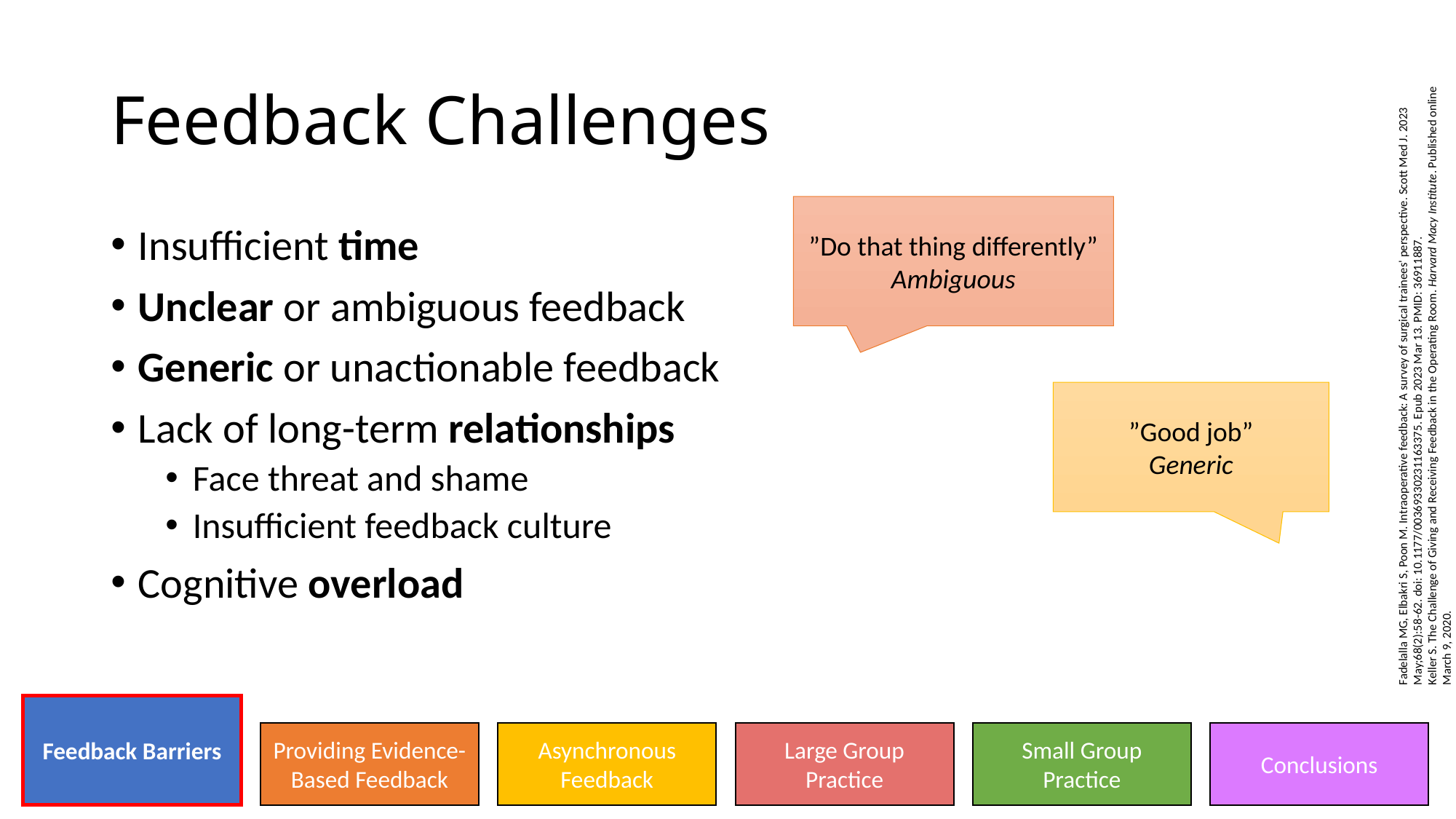

# Feedback Challenges
”Do that thing differently”
Ambiguous
Insufficient time
Unclear or ambiguous feedback
Generic or unactionable feedback
Lack of long-term relationships
Face threat and shame
Insufficient feedback culture
Cognitive overload
Fadelalla MG, Elbakri S, Poon M. Intraoperative feedback: A survey of surgical trainees' perspective. Scott Med J. 2023 May;68(2):58-62. doi: 10.1177/00369330231163375. Epub 2023 Mar 13. PMID: 36911887.
Keller S. The Challenge of Giving and Receiving Feedback in the Operating Room. Harvard Macy Institute. Published online March 9, 2020.
”Good job”
Generic
Feedback Barriers
Providing Evidence-Based Feedback
Asynchronous Feedback
Large Group Practice
Small Group Practice
Conclusions

## Slide 7
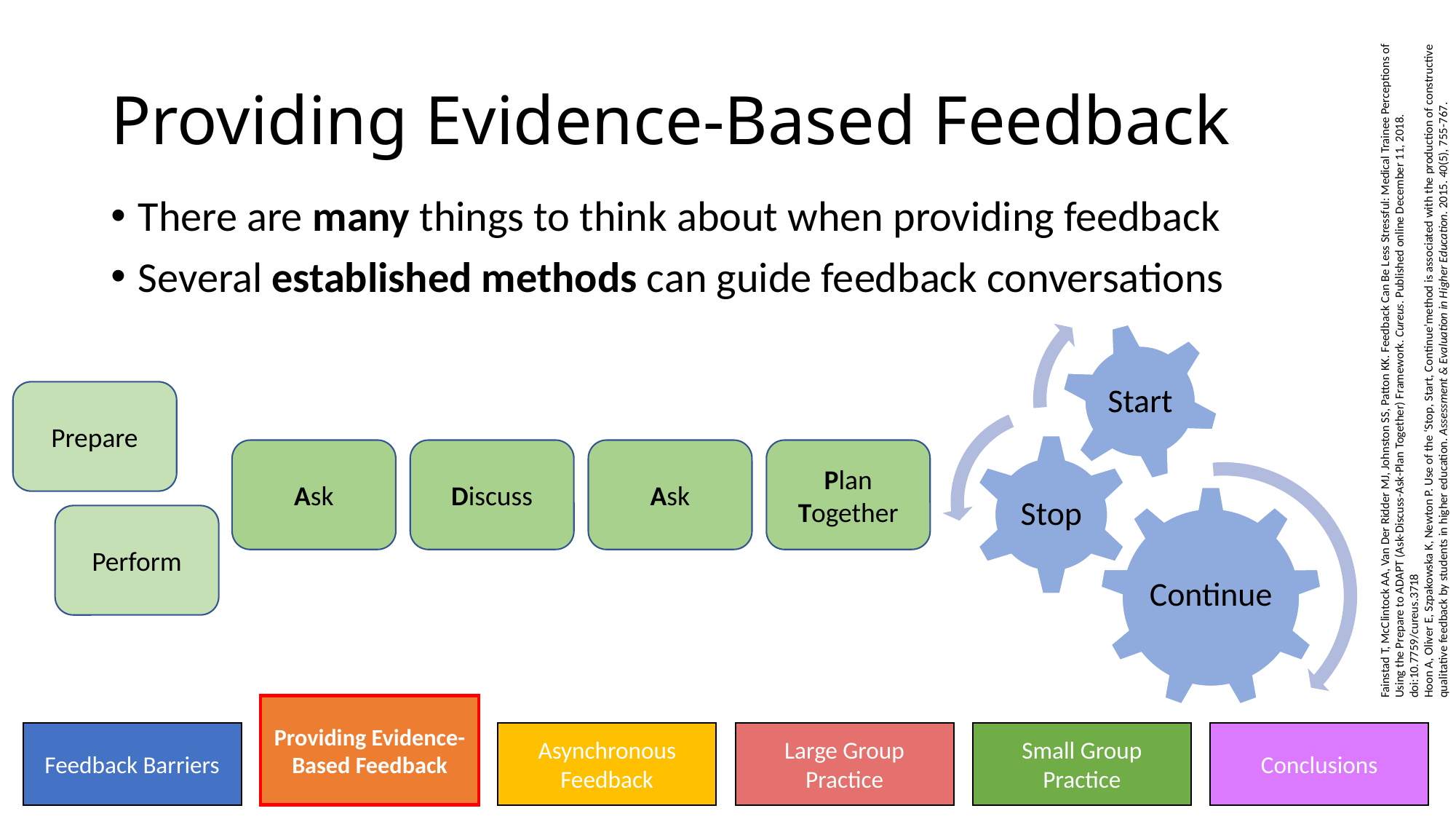

# Providing Evidence-Based Feedback
There are many things to think about when providing feedback
Several established methods can guide feedback conversations
Fainstad T, McClintock AA, Van Der Ridder MJ, Johnston SS, Patton KK. Feedback Can Be Less Stressful: Medical Trainee Perceptions of Using the Prepare to ADAPT (Ask-Discuss-Ask-Plan Together) Framework. Cureus. Published online December 11, 2018. doi:10.7759/cureus.3718
Hoon A, Oliver E, Szpakowska K, Newton P. Use of the ‘Stop, Start, Continue’method is associated with the production of constructive qualitative feedback by students in higher education. Assessment & Evaluation in Higher Education. 2015. 40(5), 755-767.
Prepare
Ask
Plan Together
Discuss
Ask
Perform
Providing Evidence-Based Feedback
Feedback Barriers
Asynchronous Feedback
Large Group Practice
Small Group Practice
Conclusions

## Slide 8
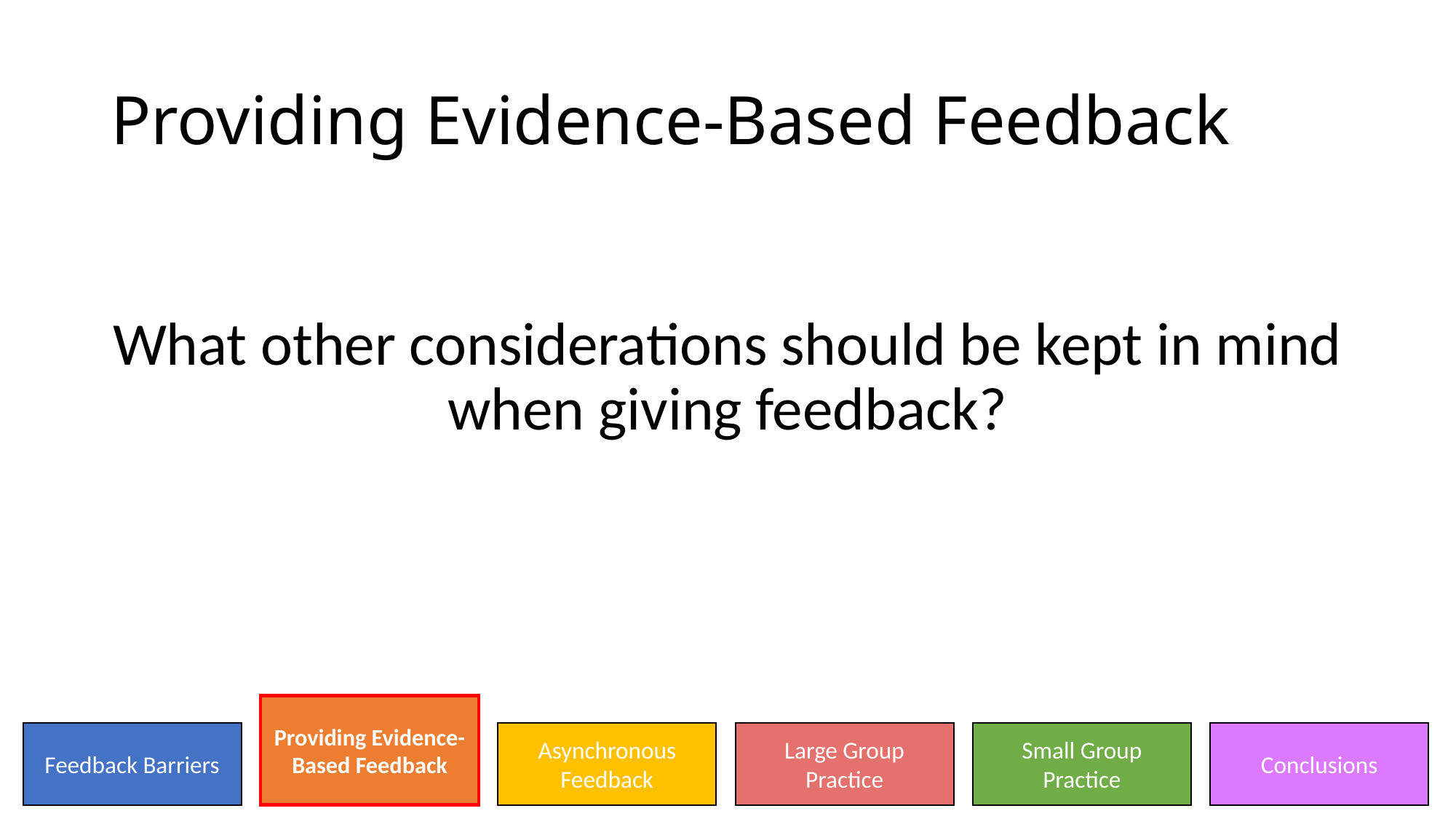

# Providing Evidence-Based Feedback
What other considerations should be kept in mind when giving feedback?
Providing Evidence-Based Feedback
Feedback Barriers
Asynchronous Feedback
Large Group Practice
Small Group Practice
Conclusions

## Slide 9
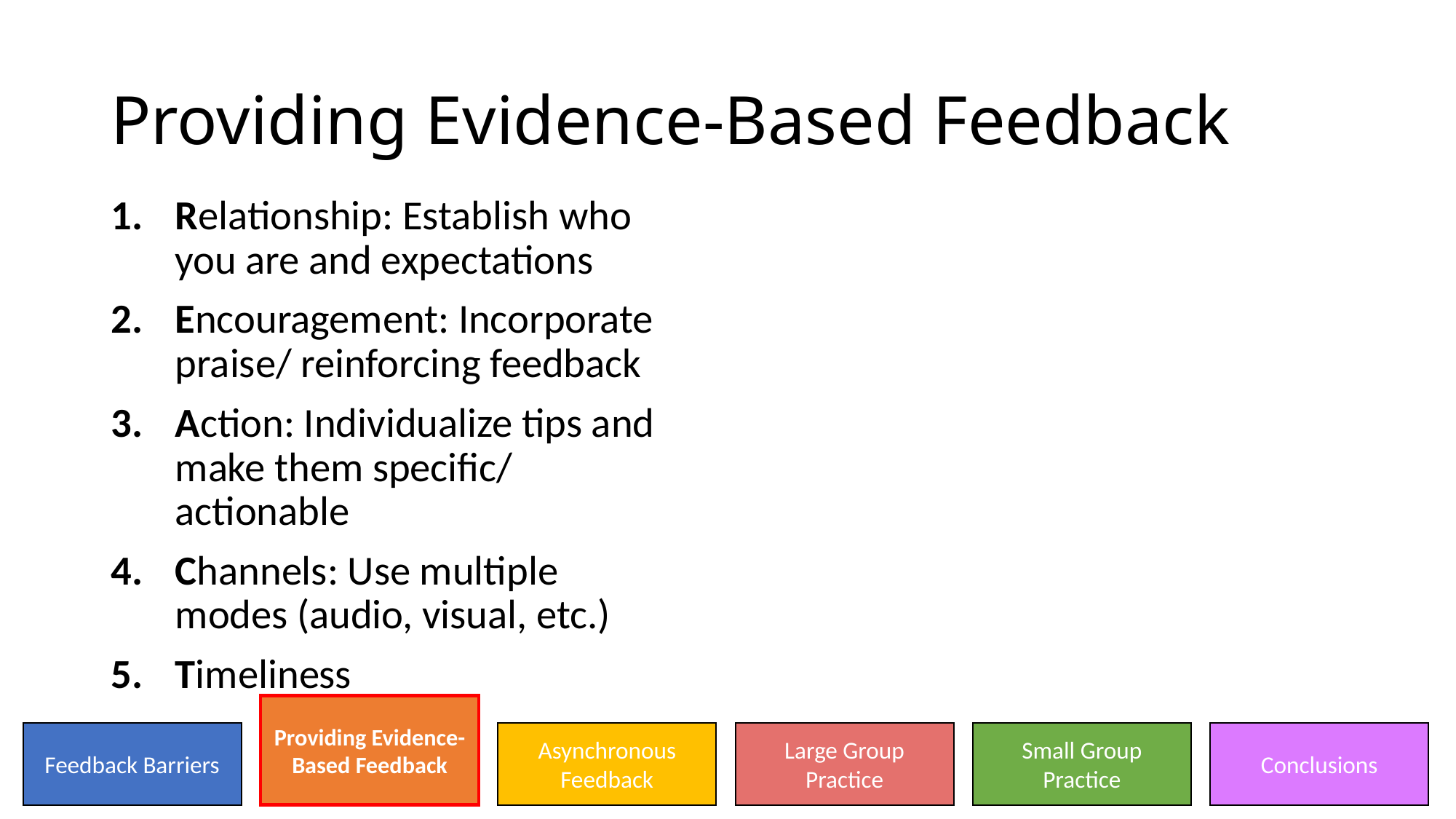

# Providing Evidence-Based Feedback
Relationship: Establish who you are and expectations
Encouragement: Incorporate praise/ reinforcing feedback
Action: Individualize tips and make them specific/ actionable
Channels: Use multiple modes (audio, visual, etc.)
Timeliness
Providing Evidence-Based Feedback
Feedback Barriers
Asynchronous Feedback
Large Group Practice
Small Group Practice
Conclusions

## Slide 10
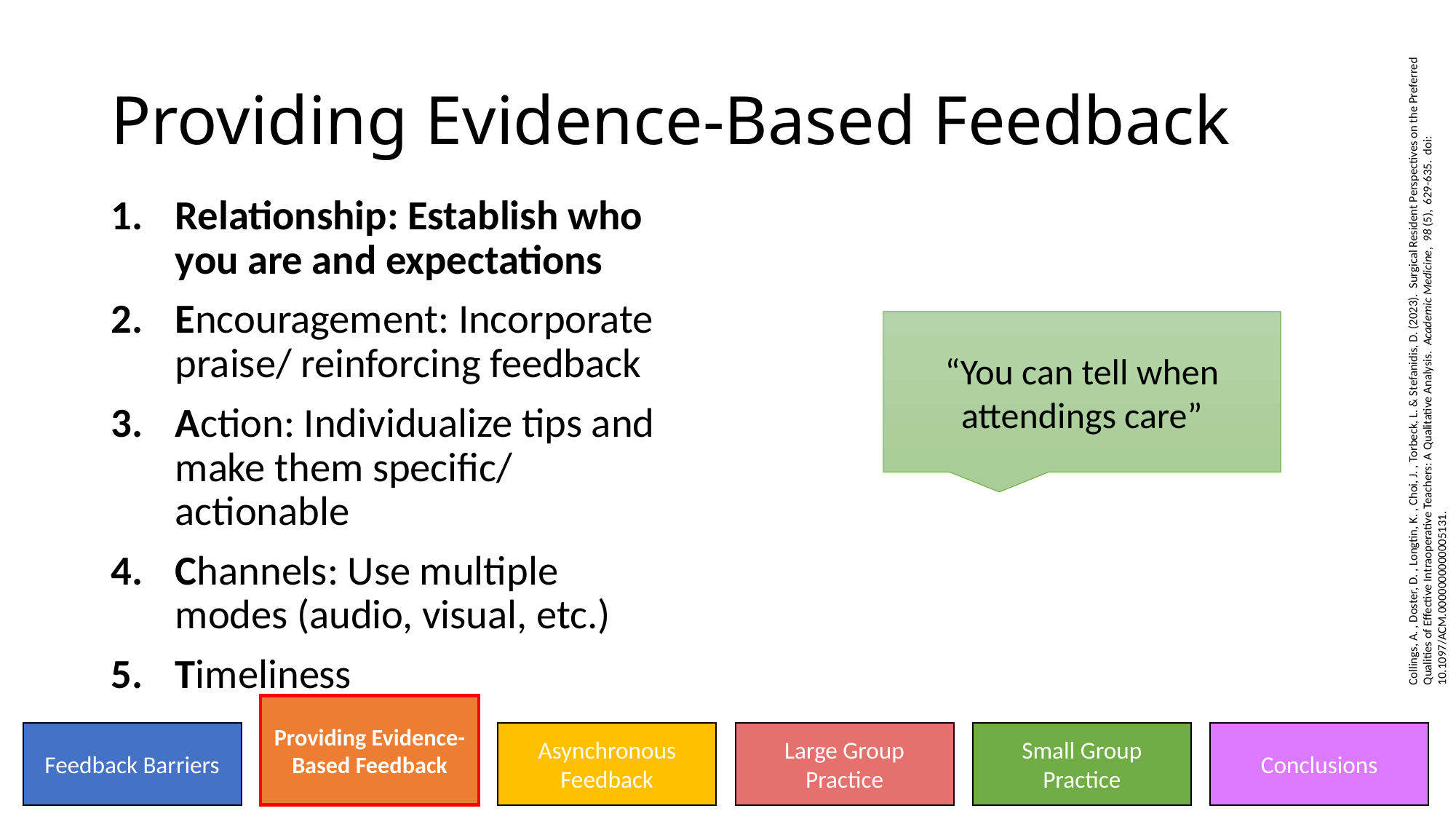

# Providing Evidence-Based Feedback
Relationship: Establish who you are and expectations
Encouragement: Incorporate praise/ reinforcing feedback
Action: Individualize tips and make them specific/ actionable
Channels: Use multiple modes (audio, visual, etc.)
Timeliness
“You can tell when attendings care”
Collings, A. , Doster, D. , Longtin, K. , Choi, J. , Torbeck, L. & Stefanidis, D. (2023).  Surgical Resident Perspectives on the Preferred Qualities of Effective Intraoperative Teachers: A Qualitative Analysis.  Academic Medicine,  98 (5),  629-635.  doi: 10.1097/ACM.0000000000005131.
Providing Evidence-Based Feedback
Feedback Barriers
Asynchronous Feedback
Large Group Practice
Small Group Practice
Conclusions

## Slide 11
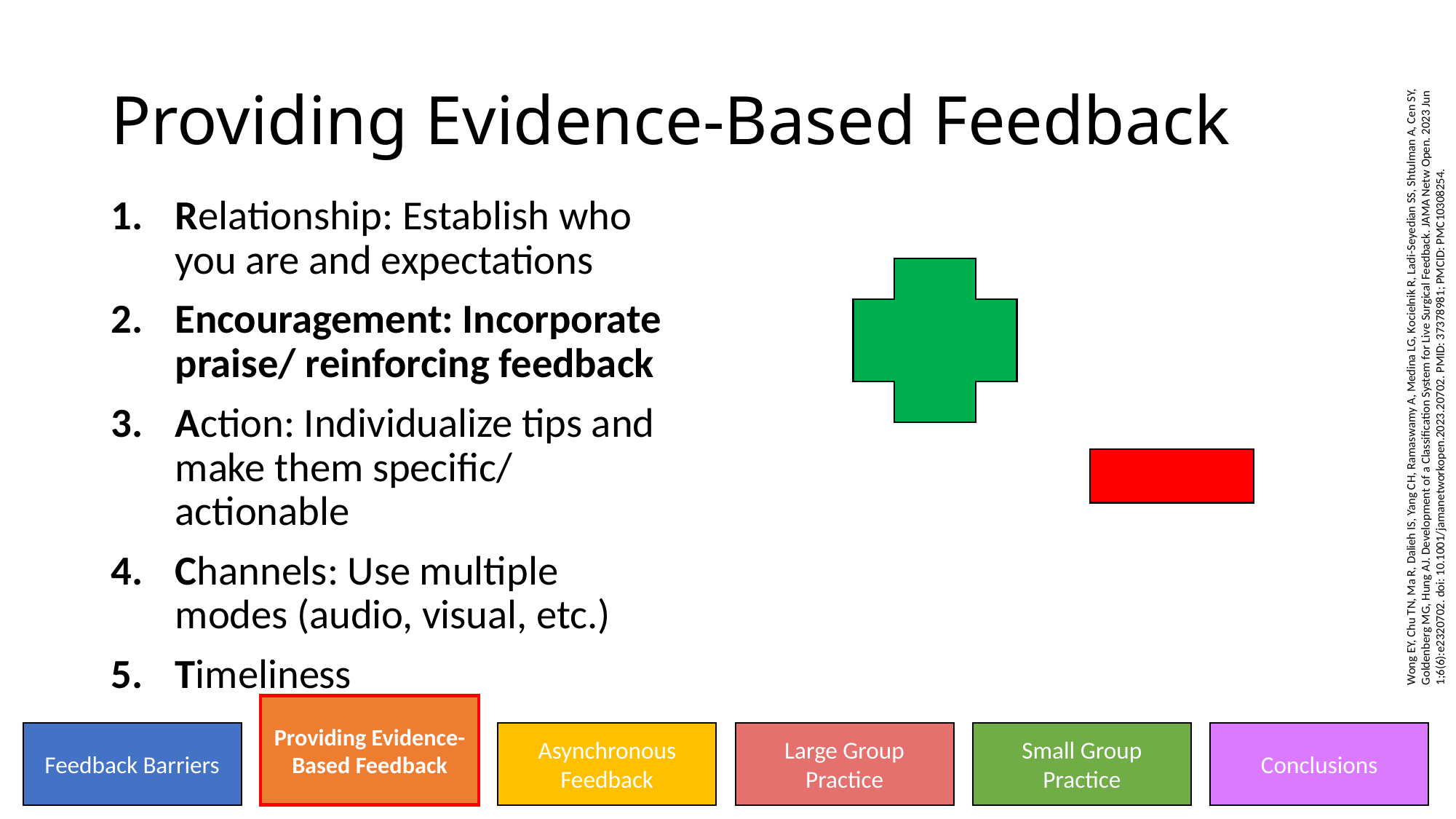

# Providing Evidence-Based Feedback
Relationship: Establish who you are and expectations
Encouragement: Incorporate praise/ reinforcing feedback
Action: Individualize tips and make them specific/ actionable
Channels: Use multiple modes (audio, visual, etc.)
Timeliness
Wong EY, Chu TN, Ma R, Dalieh IS, Yang CH, Ramaswamy A, Medina LG, Kocielnik R, Ladi-Seyedian SS, Shtulman A, Cen SY, Goldenberg MG, Hung AJ. Development of a Classification System for Live Surgical Feedback. JAMA Netw Open. 2023 Jun 1;6(6):e2320702. doi: 10.1001/jamanetworkopen.2023.20702. PMID: 37378981; PMCID: PMC10308254.
Providing Evidence-Based Feedback
Feedback Barriers
Asynchronous Feedback
Large Group Practice
Small Group Practice
Conclusions

## Slide 12
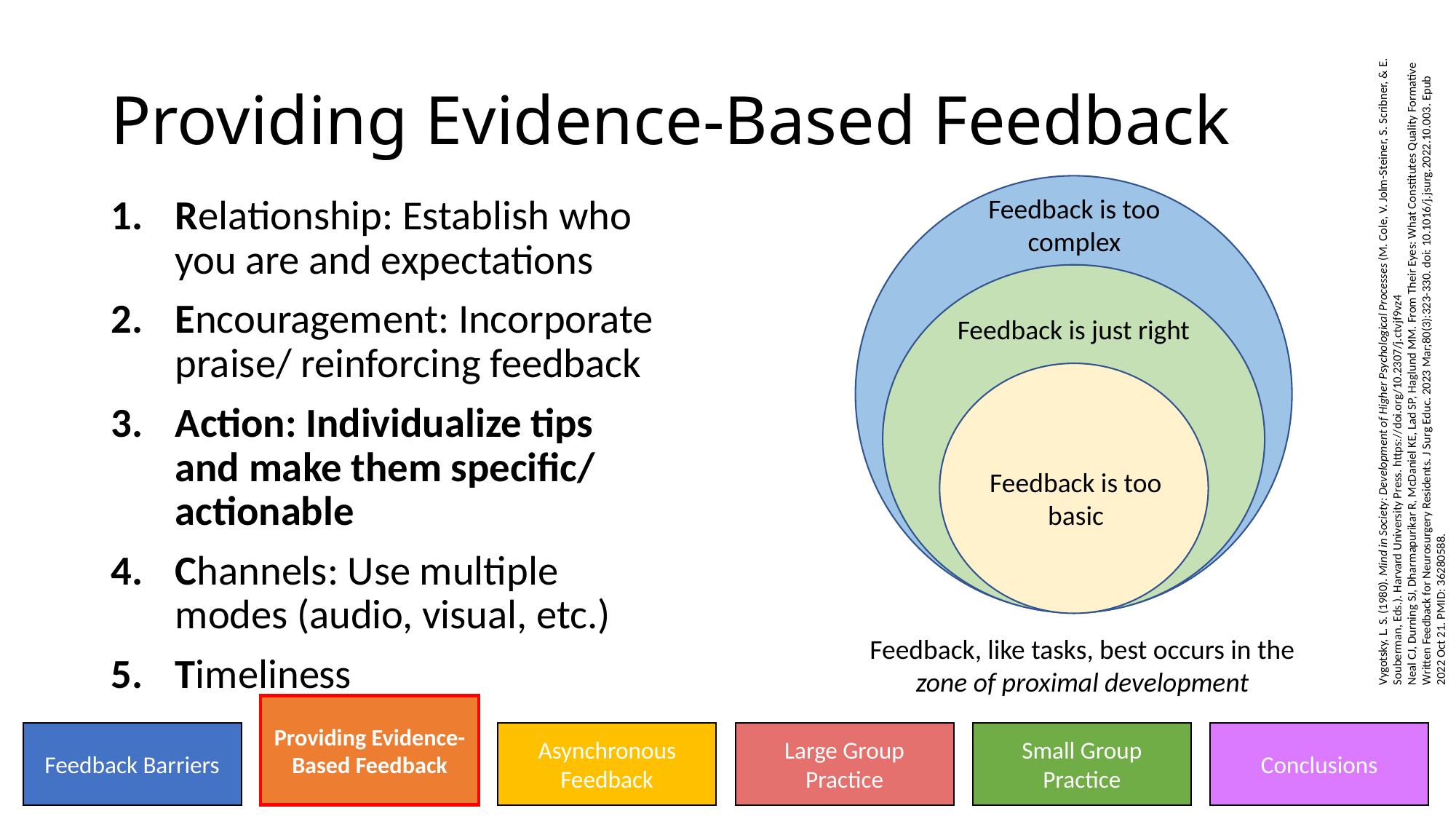

# Providing Evidence-Based Feedback
Feedback is too complex
Relationship: Establish who you are and expectations
Encouragement: Incorporate praise/ reinforcing feedback
Action: Individualize tips and make them specific/ actionable
Channels: Use multiple modes (audio, visual, etc.)
Timeliness
Feedback is just right
Vygotsky, L. S. (1980). Mind in Society: Development of Higher Psychological Processes (M. Cole, V. Jolm-Steiner, S. Scribner, & E. Souberman, Eds.). Harvard University Press. https://doi.org/10.2307/j.ctvjf9vz4
Neal CJ, Durning SJ, Dharmapurikar R, McDaniel KE, Lad SP, Haglund MM. From Their Eyes: What Constitutes Quality Formative Written Feedback for Neurosurgery Residents. J Surg Educ. 2023 Mar;80(3):323-330. doi: 10.1016/j.jsurg.2022.10.003. Epub 2022 Oct 21. PMID: 36280588.
Feedback is too basic
Feedback, like tasks, best occurs in the zone of proximal development
Providing Evidence-Based Feedback
Feedback Barriers
Asynchronous Feedback
Large Group Practice
Small Group Practice
Conclusions

## Slide 13
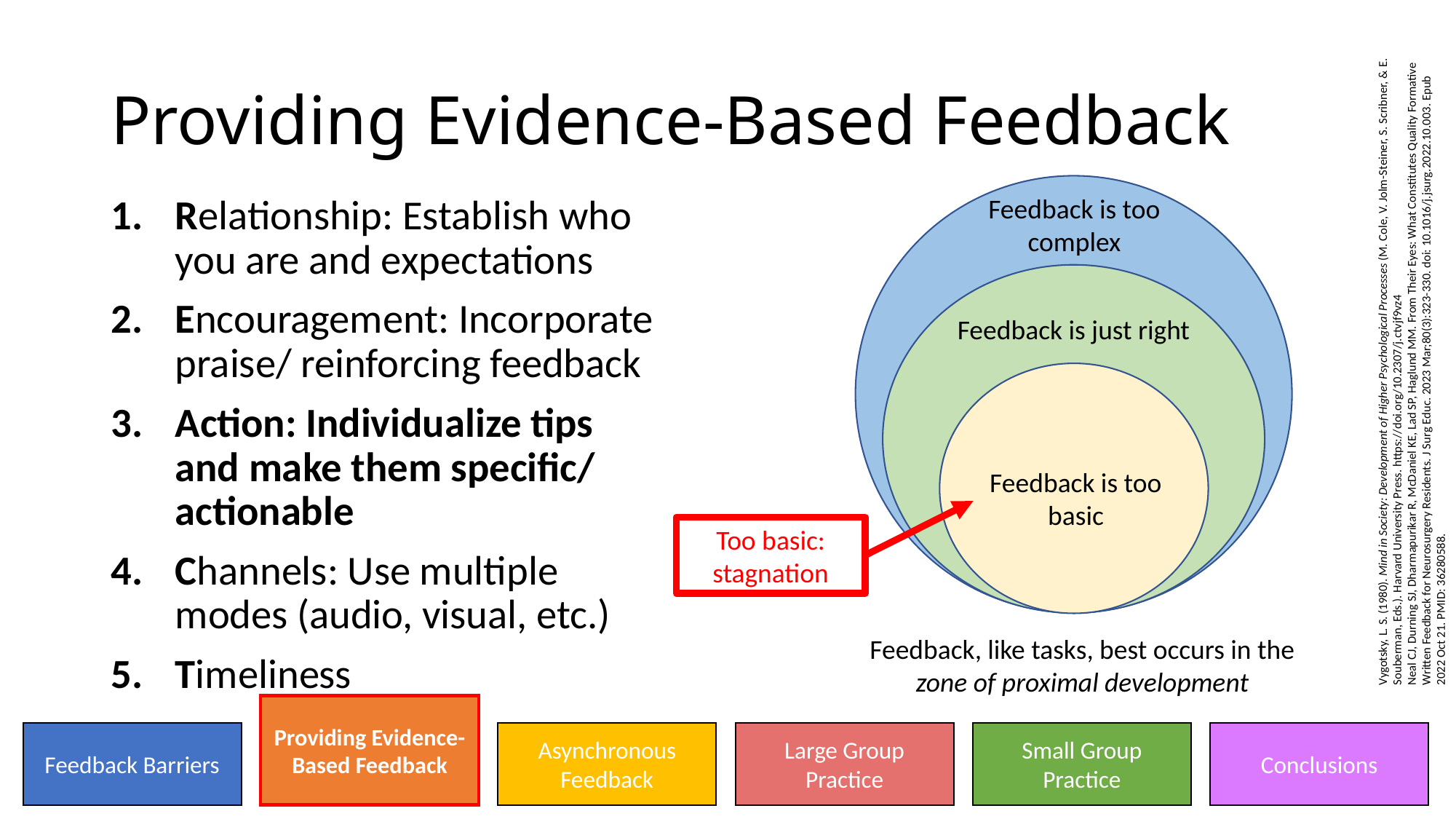

# Providing Evidence-Based Feedback
Feedback is too complex
Relationship: Establish who you are and expectations
Encouragement: Incorporate praise/ reinforcing feedback
Action: Individualize tips and make them specific/ actionable
Channels: Use multiple modes (audio, visual, etc.)
Timeliness
Feedback is just right
Vygotsky, L. S. (1980). Mind in Society: Development of Higher Psychological Processes (M. Cole, V. Jolm-Steiner, S. Scribner, & E. Souberman, Eds.). Harvard University Press. https://doi.org/10.2307/j.ctvjf9vz4
Neal CJ, Durning SJ, Dharmapurikar R, McDaniel KE, Lad SP, Haglund MM. From Their Eyes: What Constitutes Quality Formative Written Feedback for Neurosurgery Residents. J Surg Educ. 2023 Mar;80(3):323-330. doi: 10.1016/j.jsurg.2022.10.003. Epub 2022 Oct 21. PMID: 36280588.
Feedback is too basic
Too basic: stagnation
Feedback, like tasks, best occurs in the zone of proximal development
Providing Evidence-Based Feedback
Feedback Barriers
Asynchronous Feedback
Large Group Practice
Small Group Practice
Conclusions

## Slide 14
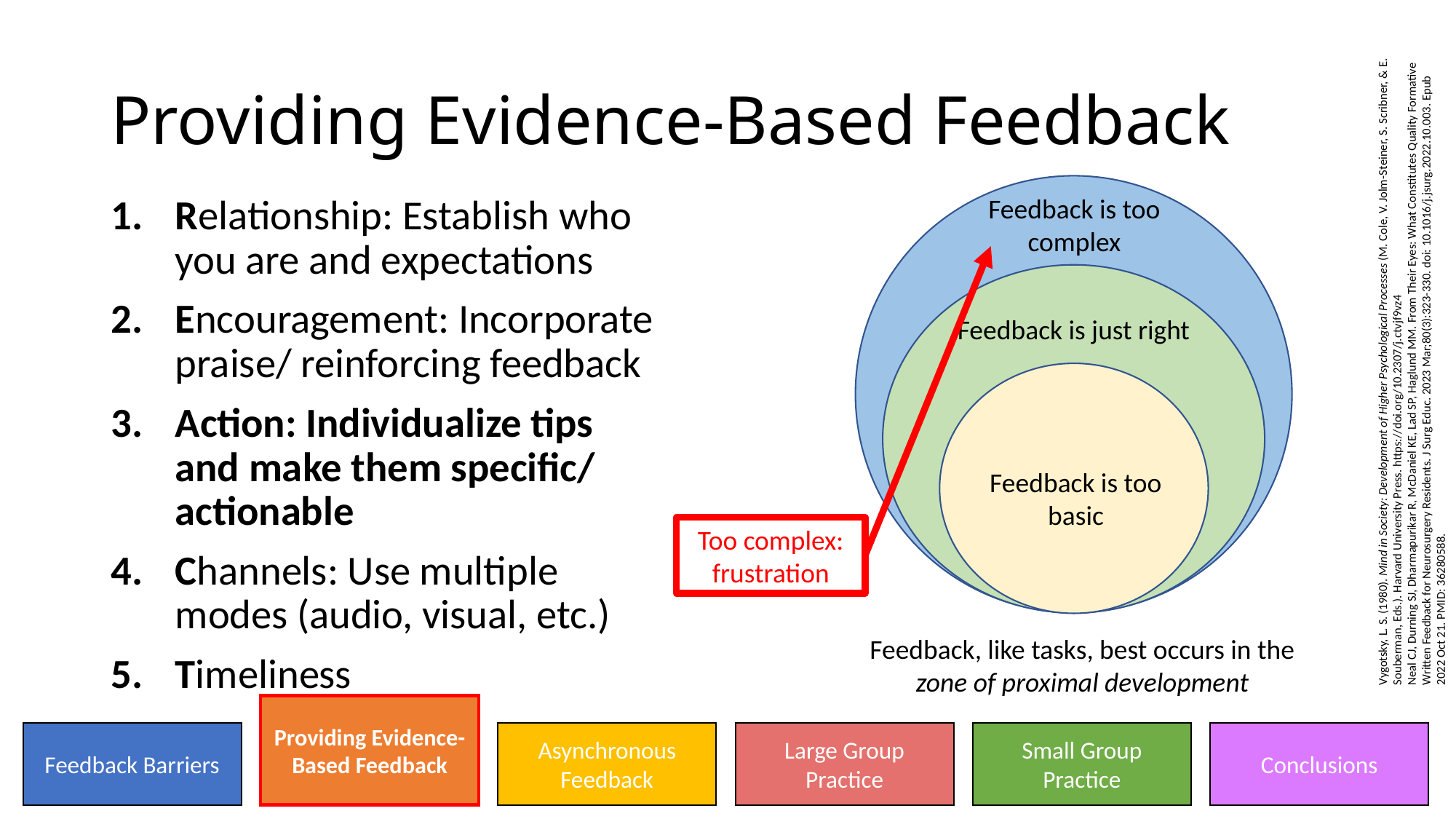

# Providing Evidence-Based Feedback
Feedback is too complex
Relationship: Establish who you are and expectations
Encouragement: Incorporate praise/ reinforcing feedback
Action: Individualize tips and make them specific/ actionable
Channels: Use multiple modes (audio, visual, etc.)
Timeliness
Feedback is just right
Vygotsky, L. S. (1980). Mind in Society: Development of Higher Psychological Processes (M. Cole, V. Jolm-Steiner, S. Scribner, & E. Souberman, Eds.). Harvard University Press. https://doi.org/10.2307/j.ctvjf9vz4
Neal CJ, Durning SJ, Dharmapurikar R, McDaniel KE, Lad SP, Haglund MM. From Their Eyes: What Constitutes Quality Formative Written Feedback for Neurosurgery Residents. J Surg Educ. 2023 Mar;80(3):323-330. doi: 10.1016/j.jsurg.2022.10.003. Epub 2022 Oct 21. PMID: 36280588.
Feedback is too basic
Too complex: frustration
Feedback, like tasks, best occurs in the zone of proximal development
Providing Evidence-Based Feedback
Feedback Barriers
Asynchronous Feedback
Large Group Practice
Small Group Practice
Conclusions

## Slide 15
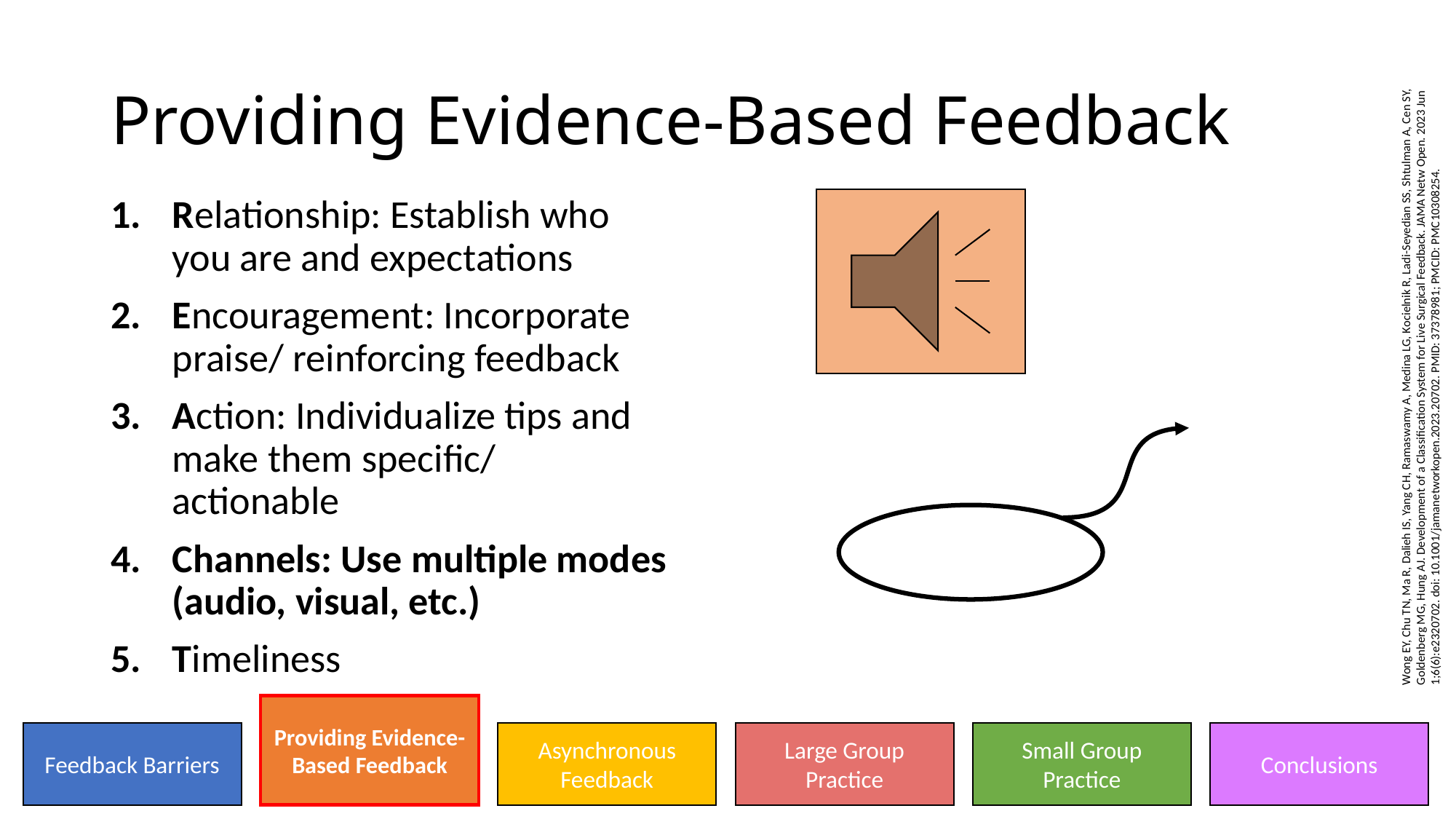

# Providing Evidence-Based Feedback
Relationship: Establish who you are and expectations
Encouragement: Incorporate praise/ reinforcing feedback
Action: Individualize tips and make them specific/ actionable
Channels: Use multiple modes (audio, visual, etc.)
Timeliness
Wong EY, Chu TN, Ma R, Dalieh IS, Yang CH, Ramaswamy A, Medina LG, Kocielnik R, Ladi-Seyedian SS, Shtulman A, Cen SY, Goldenberg MG, Hung AJ. Development of a Classification System for Live Surgical Feedback. JAMA Netw Open. 2023 Jun 1;6(6):e2320702. doi: 10.1001/jamanetworkopen.2023.20702. PMID: 37378981; PMCID: PMC10308254.
Providing Evidence-Based Feedback
Feedback Barriers
Asynchronous Feedback
Large Group Practice
Small Group Practice
Conclusions

## Slide 16
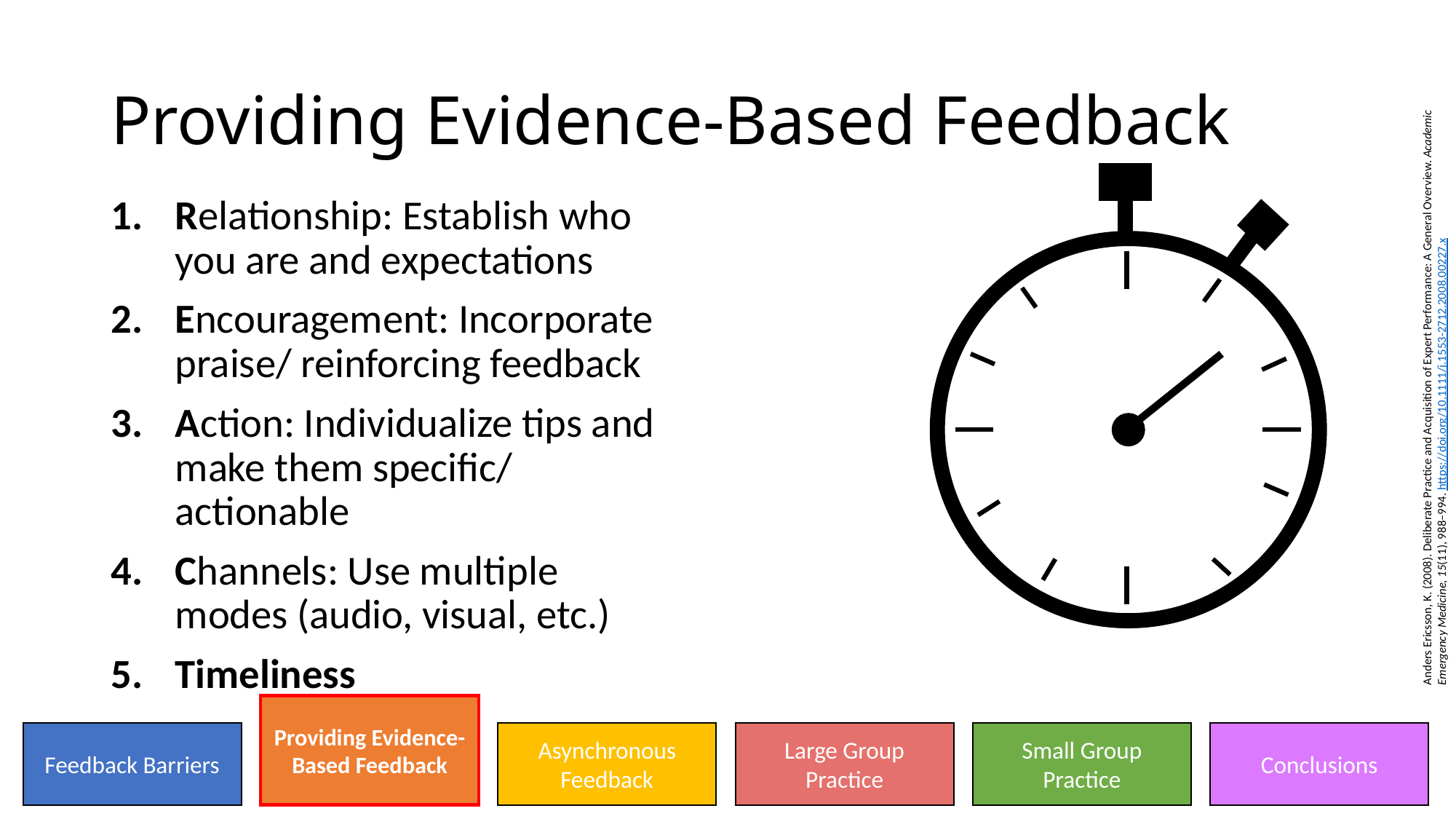

# Providing Evidence-Based Feedback
Relationship: Establish who you are and expectations
Encouragement: Incorporate praise/ reinforcing feedback
Action: Individualize tips and make them specific/ actionable
Channels: Use multiple modes (audio, visual, etc.)
Timeliness
Anders Ericsson, K. (2008). Deliberate Practice and Acquisition of Expert Performance: A General Overview. Academic Emergency Medicine, 15(11), 988–994. https://doi.org/10.1111/j.1553-2712.2008.00227.x
Providing Evidence-Based Feedback
Feedback Barriers
Asynchronous Feedback
Large Group Practice
Small Group Practice
Conclusions

## Slide 17
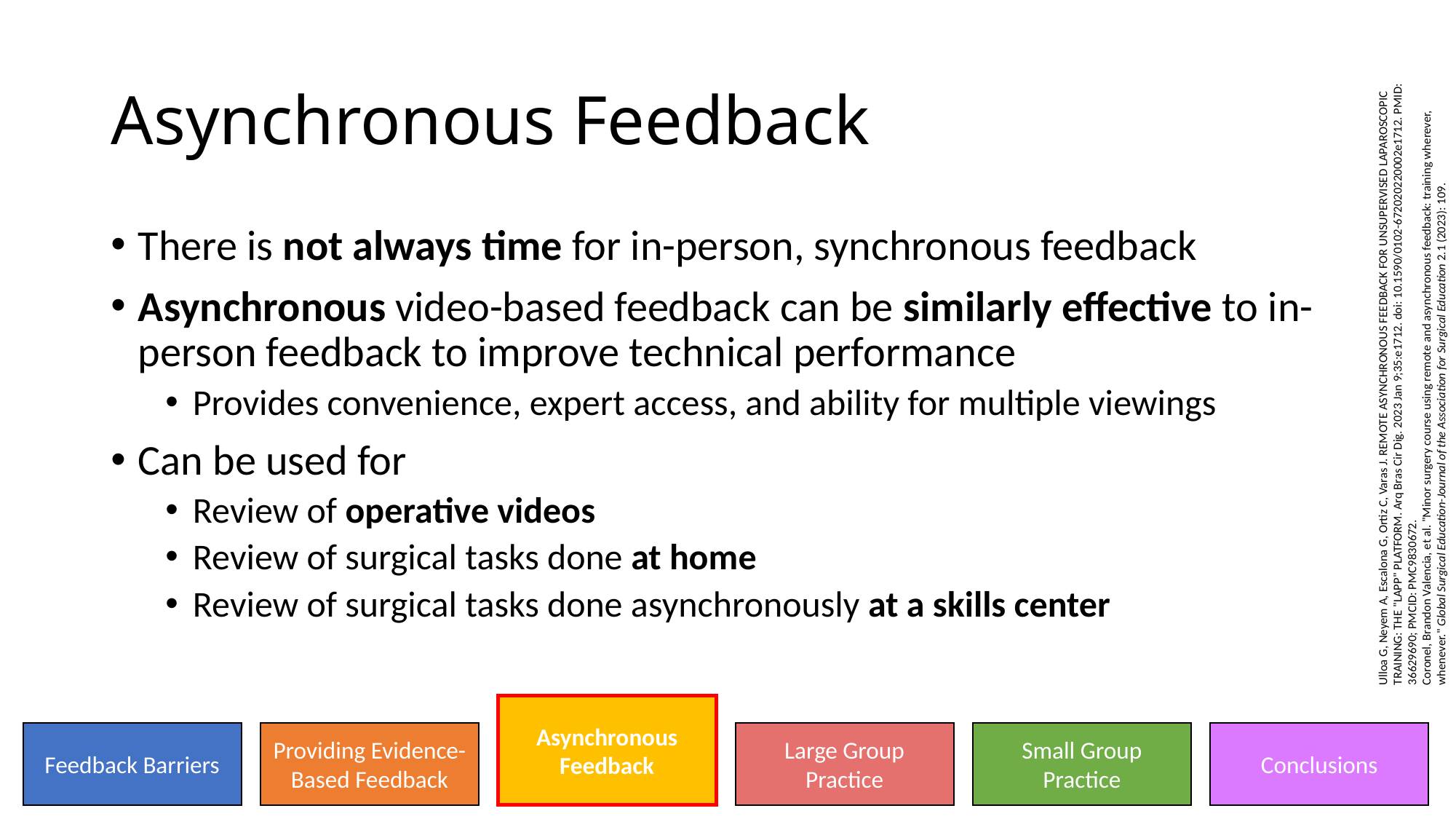

# Asynchronous Feedback
There is not always time for in-person, synchronous feedback
Asynchronous video-based feedback can be similarly effective to in-person feedback to improve technical performance
Provides convenience, expert access, and ability for multiple viewings
Can be used for
Review of operative videos
Review of surgical tasks done at home
Review of surgical tasks done asynchronously at a skills center
Ulloa G, Neyem A, Escalona G, Ortiz C, Varas J. REMOTE ASYNCHRONOUS FEEDBACK FOR UNSUPERVISED LAPAROSCOPIC TRAINING: THE "LAPP" PLATFORM. Arq Bras Cir Dig. 2023 Jan 9;35:e1712. doi: 10.1590/0102-672020220002e1712. PMID: 36629690; PMCID: PMC9830672.
Coronel, Brandon Valencia, et al. "Minor surgery course using remote and asynchronous feedback: training wherever, whenever." Global Surgical Education-Journal of the Association for Surgical Education 2.1 (2023): 109.
Asynchronous Feedback
Feedback Barriers
Providing Evidence-Based Feedback
Large Group Practice
Small Group Practice
Conclusions

## Slide 18
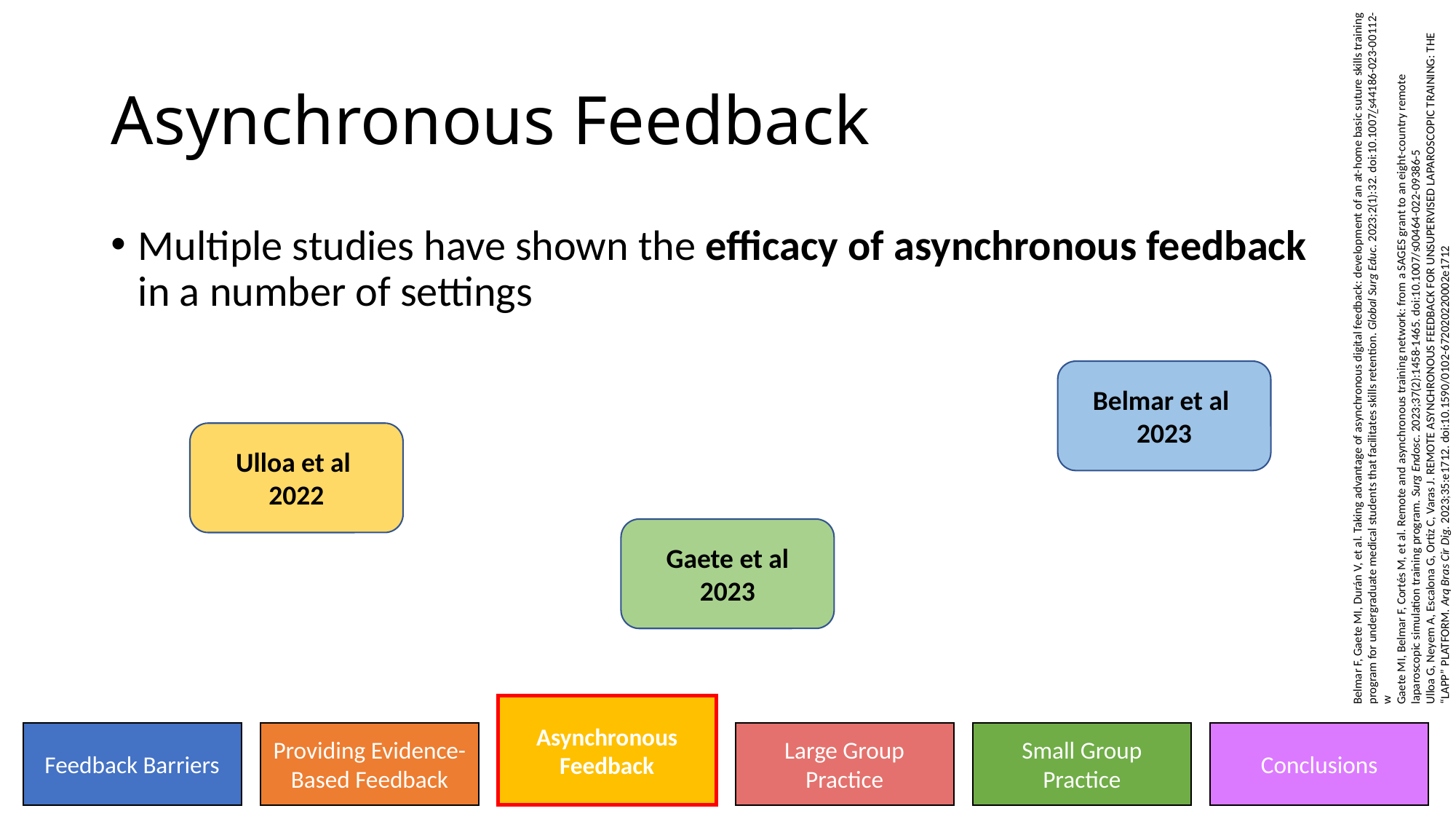

# Asynchronous Feedback
Multiple studies have shown the efficacy of asynchronous feedback in a number of settings
Belmar F, Gaete MI, Durán V, et al. Taking advantage of asynchronous digital feedback: development of an at-home basic suture skills training program for undergraduate medical students that facilitates skills retention. Global Surg Educ. 2023;2(1):32. doi:10.1007/s44186-023-00112-w
Gaete MI, Belmar F, Cortés M, et al. Remote and asynchronous training network: from a SAGES grant to an eight-country remote laparoscopic simulation training program. Surg Endosc. 2023;37(2):1458-1465. doi:10.1007/s00464-022-09386-5
Ulloa G, Neyem A, Escalona G, Ortiz C, Varas J. REMOTE ASYNCHRONOUS FEEDBACK FOR UNSUPERVISED LAPAROSCOPIC TRAINING: THE “LAPP” PLATFORM. Arq Bras Cir Dig. 2023;35:e1712. doi:10.1590/0102-672020220002e1712
Belmar et al
2023
Ulloa et al
2022
Gaete et al 2023
Asynchronous Feedback
Feedback Barriers
Providing Evidence-Based Feedback
Large Group Practice
Small Group Practice
Conclusions

## Slide 19
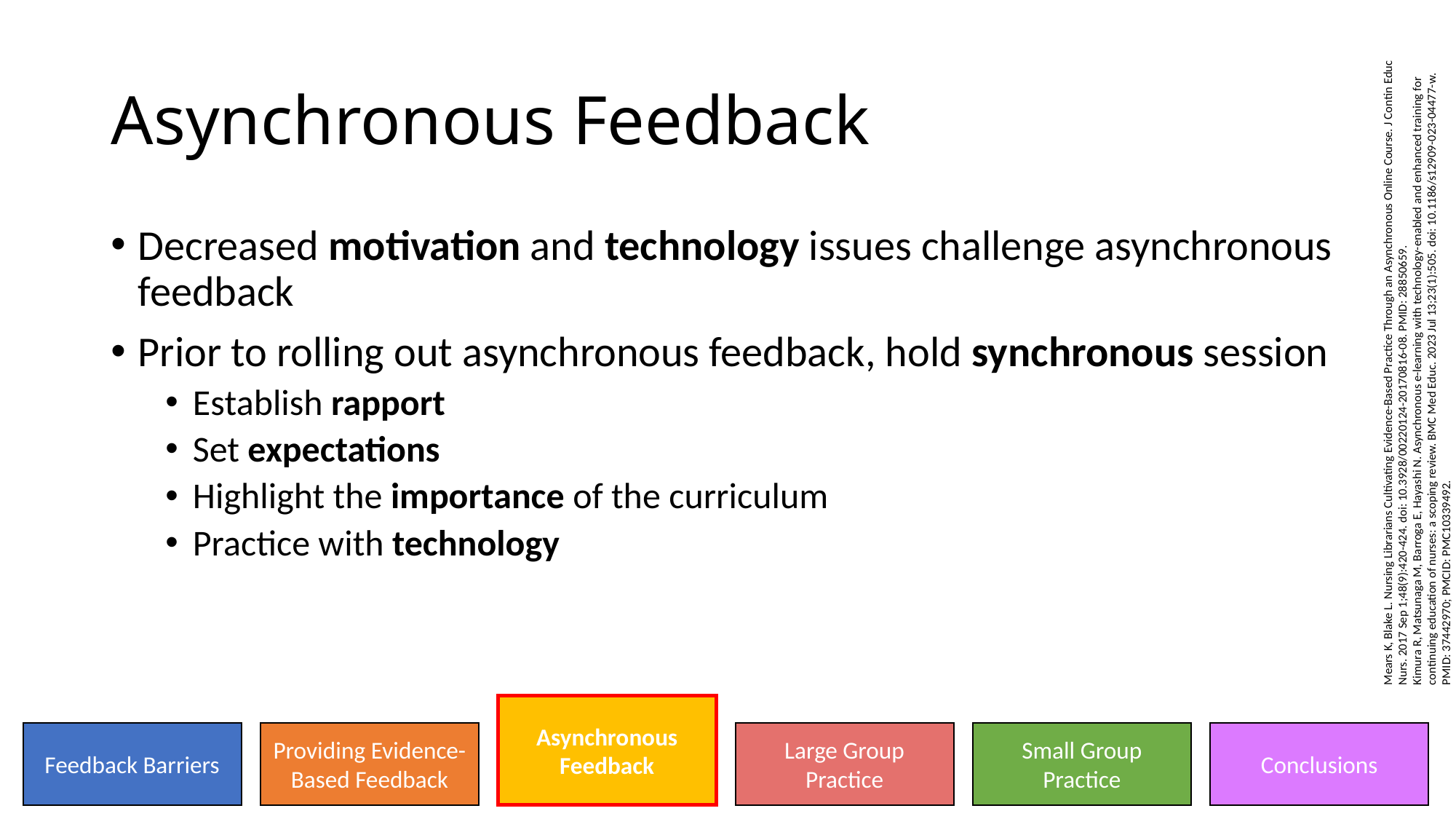

# Asynchronous Feedback
Decreased motivation and technology issues challenge asynchronous feedback
Prior to rolling out asynchronous feedback, hold synchronous session
Establish rapport
Set expectations
Highlight the importance of the curriculum
Practice with technology
Mears K, Blake L. Nursing Librarians Cultivating Evidence-Based Practice Through an Asynchronous Online Course. J Contin Educ Nurs. 2017 Sep 1;48(9):420-424. doi: 10.3928/00220124-20170816-08. PMID: 28850659.
Kimura R, Matsunaga M, Barroga E, Hayashi N. Asynchronous e-learning with technology-enabled and enhanced training for continuing education of nurses: a scoping review. BMC Med Educ. 2023 Jul 13;23(1):505. doi: 10.1186/s12909-023-04477-w. PMID: 37442970; PMCID: PMC10339492.
Asynchronous Feedback
Feedback Barriers
Providing Evidence-Based Feedback
Large Group Practice
Small Group Practice
Conclusions

## Slide 20
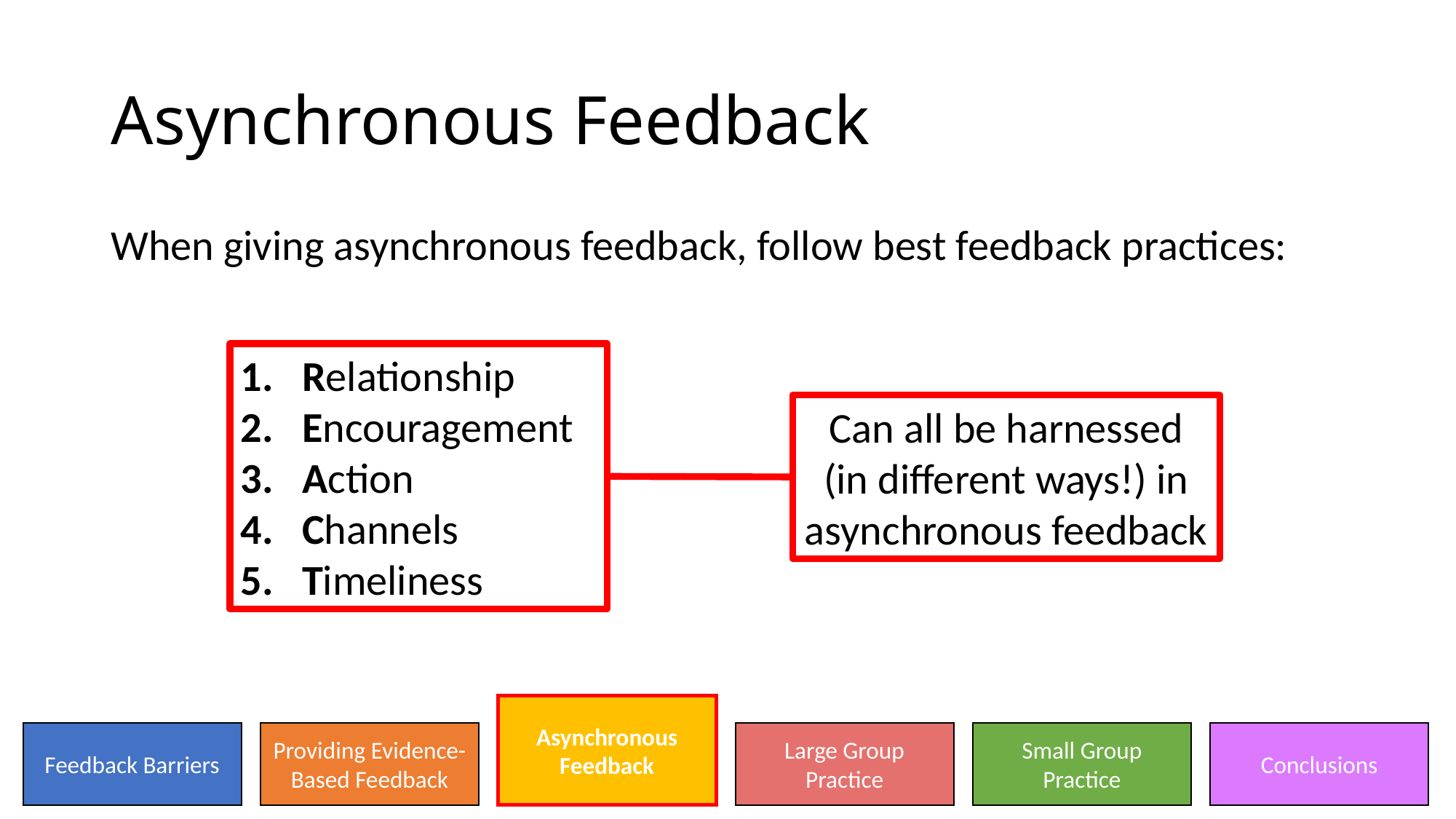

# Asynchronous Feedback
When giving asynchronous feedback, follow best feedback practices:
Relationship
Encouragement
Action
Channels
Timeliness
Can all be harnessed (in different ways!) in asynchronous feedback
Asynchronous Feedback
Feedback Barriers
Providing Evidence-Based Feedback
Large Group Practice
Small Group Practice
Conclusions

## Slide 21
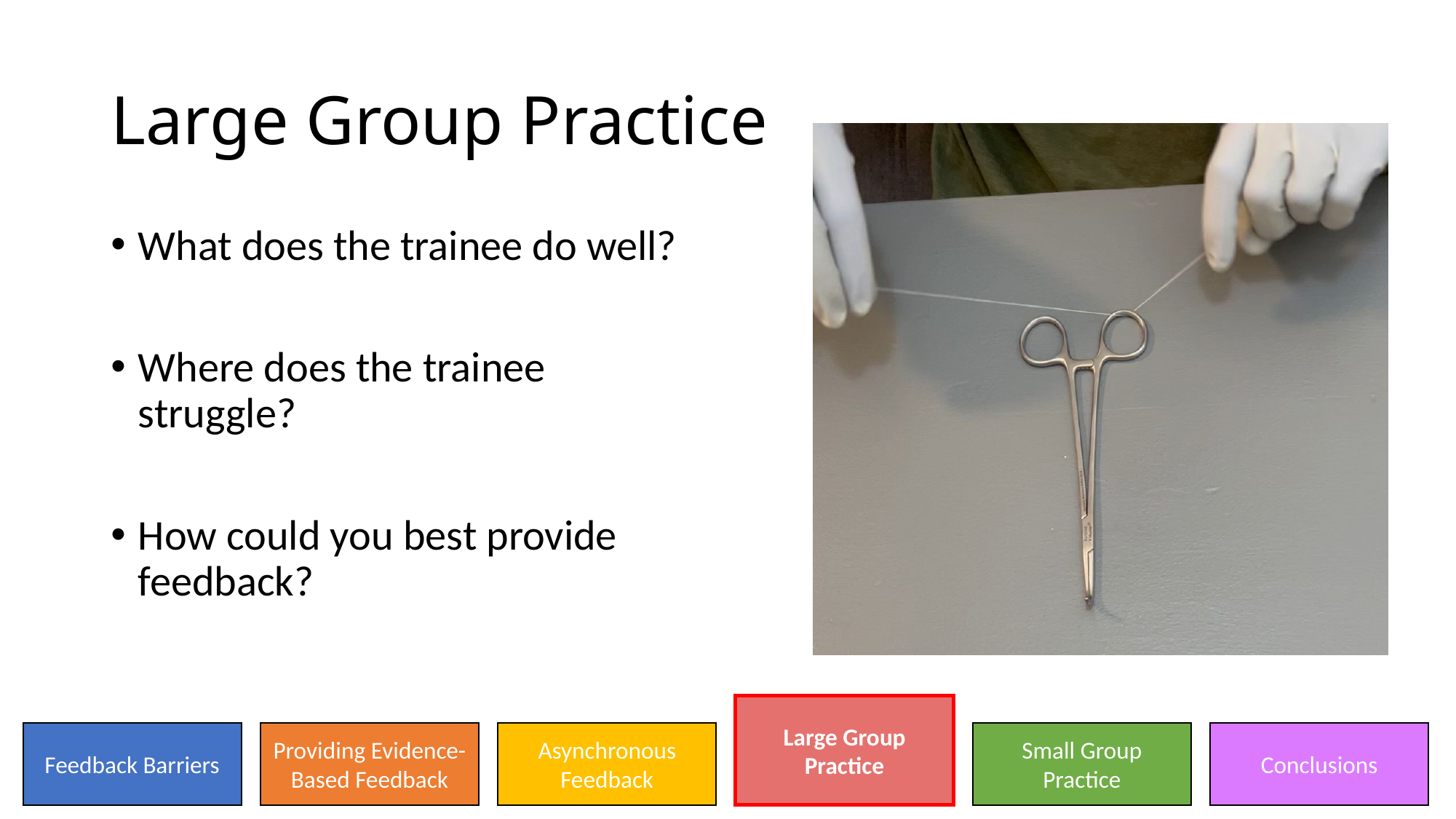

# Large Group Practice
What does the trainee do well?
Where does the trainee struggle?
How could you best provide feedback?
Large Group Practice
Feedback Barriers
Providing Evidence-Based Feedback
Asynchronous Feedback
Small Group Practice
Conclusions

## Slide 22
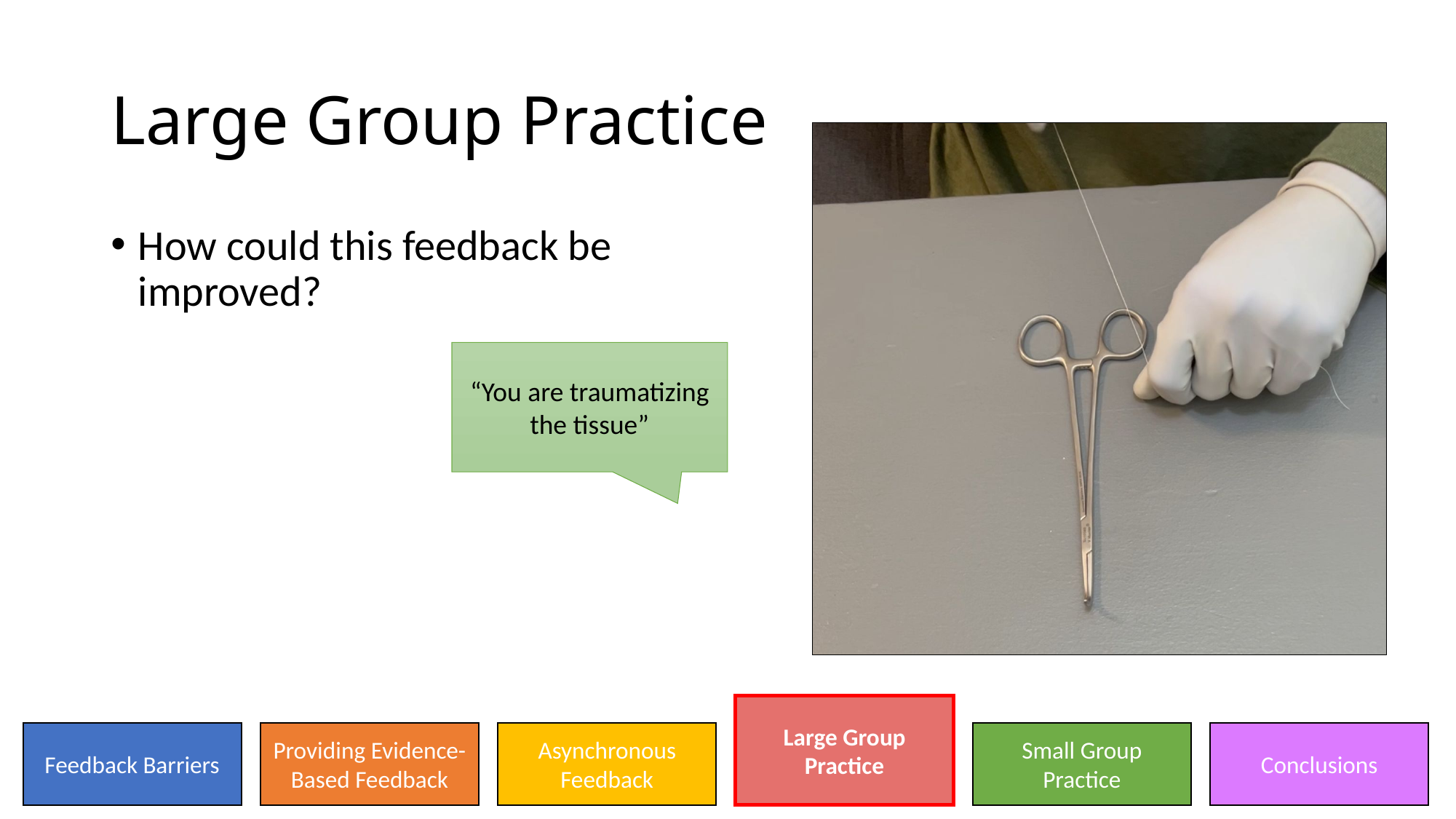

# Large Group Practice
How could this feedback be improved?
“You are traumatizing the tissue”
Large Group Practice
Feedback Barriers
Providing Evidence-Based Feedback
Asynchronous Feedback
Small Group Practice
Conclusions

## Slide 23
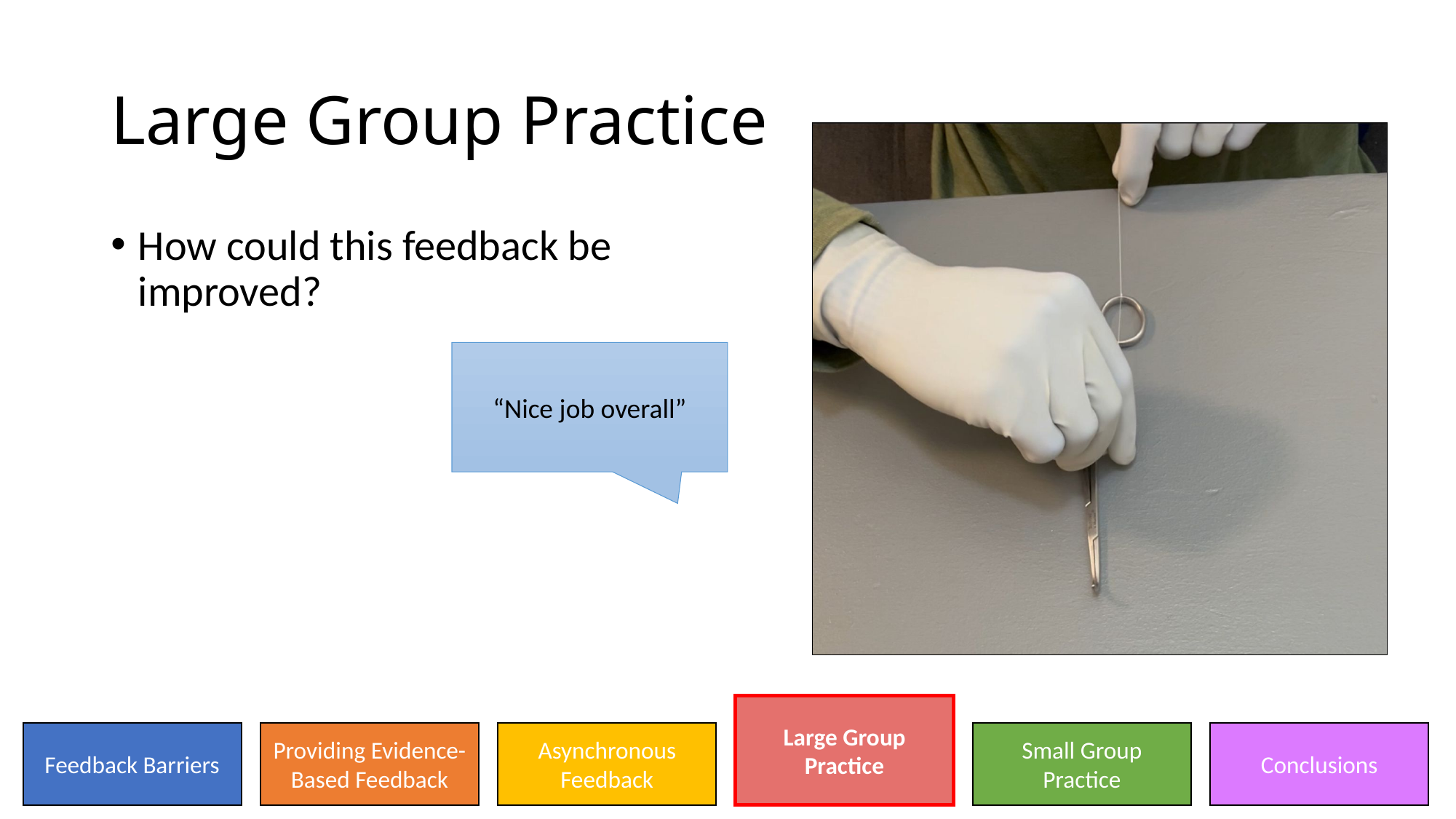

# Large Group Practice
How could this feedback be improved?
“Nice job overall”
Large Group Practice
Feedback Barriers
Providing Evidence-Based Feedback
Asynchronous Feedback
Small Group Practice
Conclusions

## Slide 24
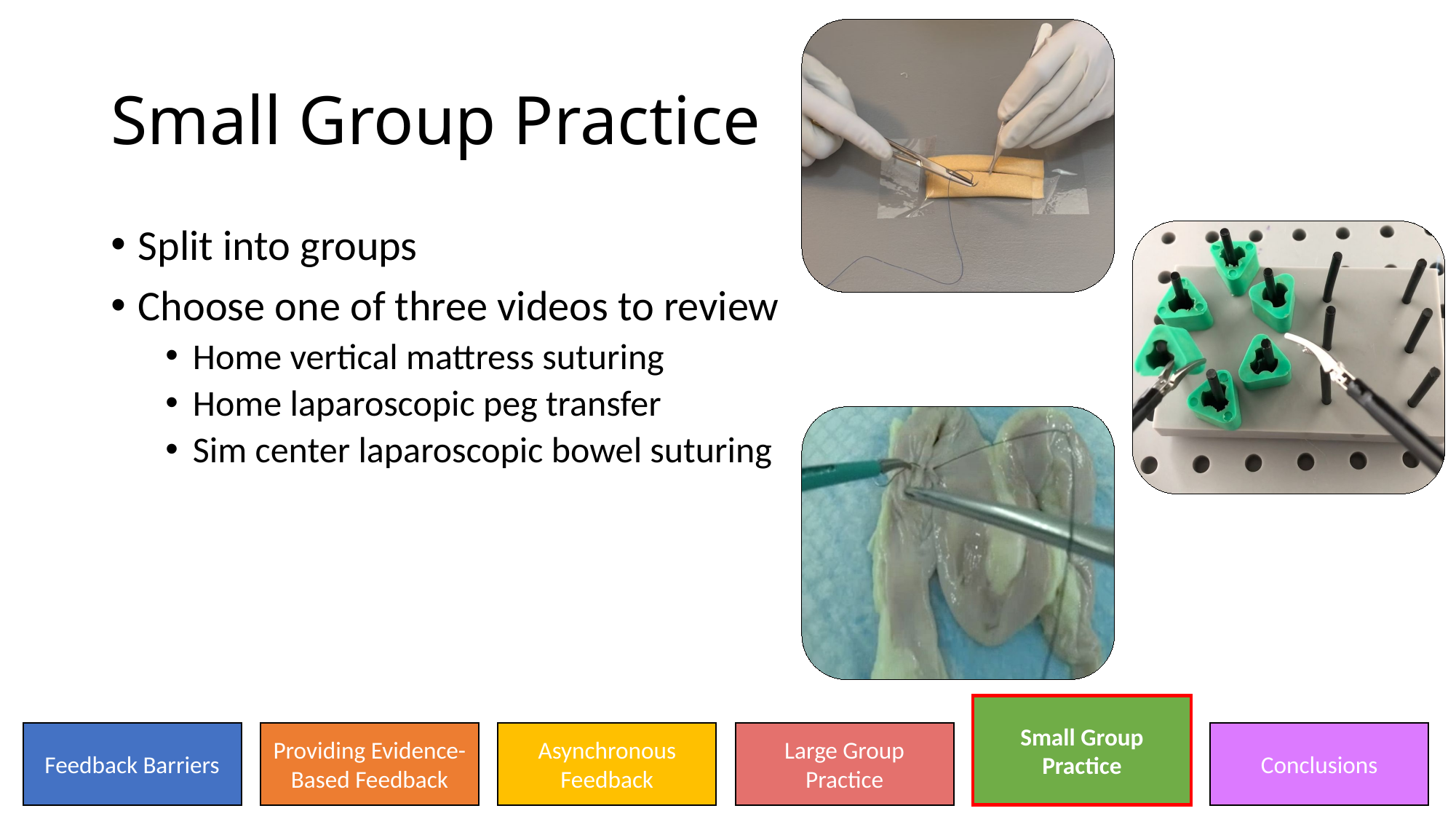

# Small Group Practice
Split into groups
Choose one of three videos to review
Home vertical mattress suturing
Home laparoscopic peg transfer
Sim center laparoscopic bowel suturing
Small Group Practice
Feedback Barriers
Providing Evidence-Based Feedback
Asynchronous Feedback
Large Group Practice
Conclusions

## Slide 25
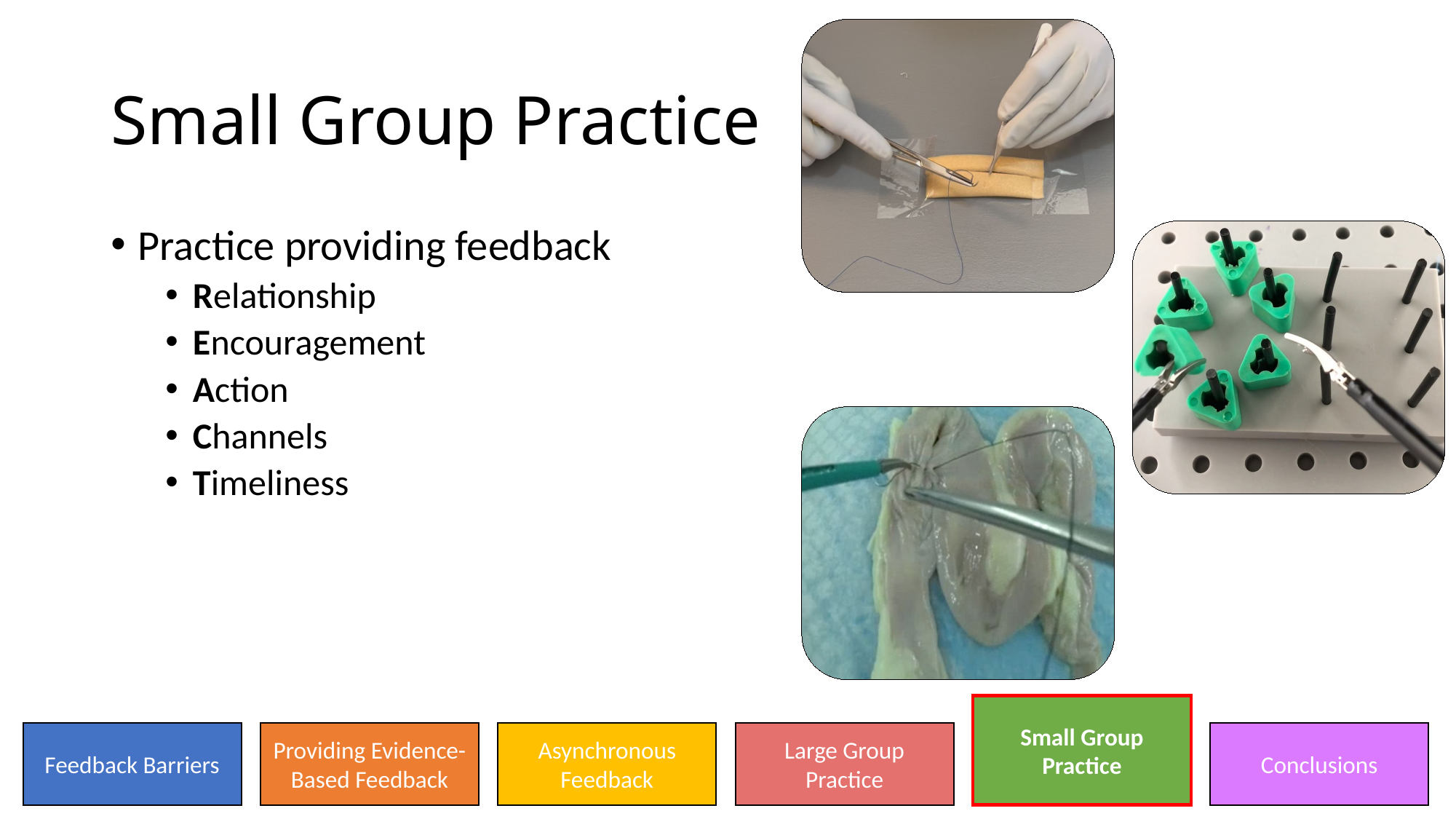

# Small Group Practice
Practice providing feedback
Relationship
Encouragement
Action
Channels
Timeliness
Small Group Practice
Feedback Barriers
Providing Evidence-Based Feedback
Asynchronous Feedback
Large Group Practice
Conclusions

## Slide 26
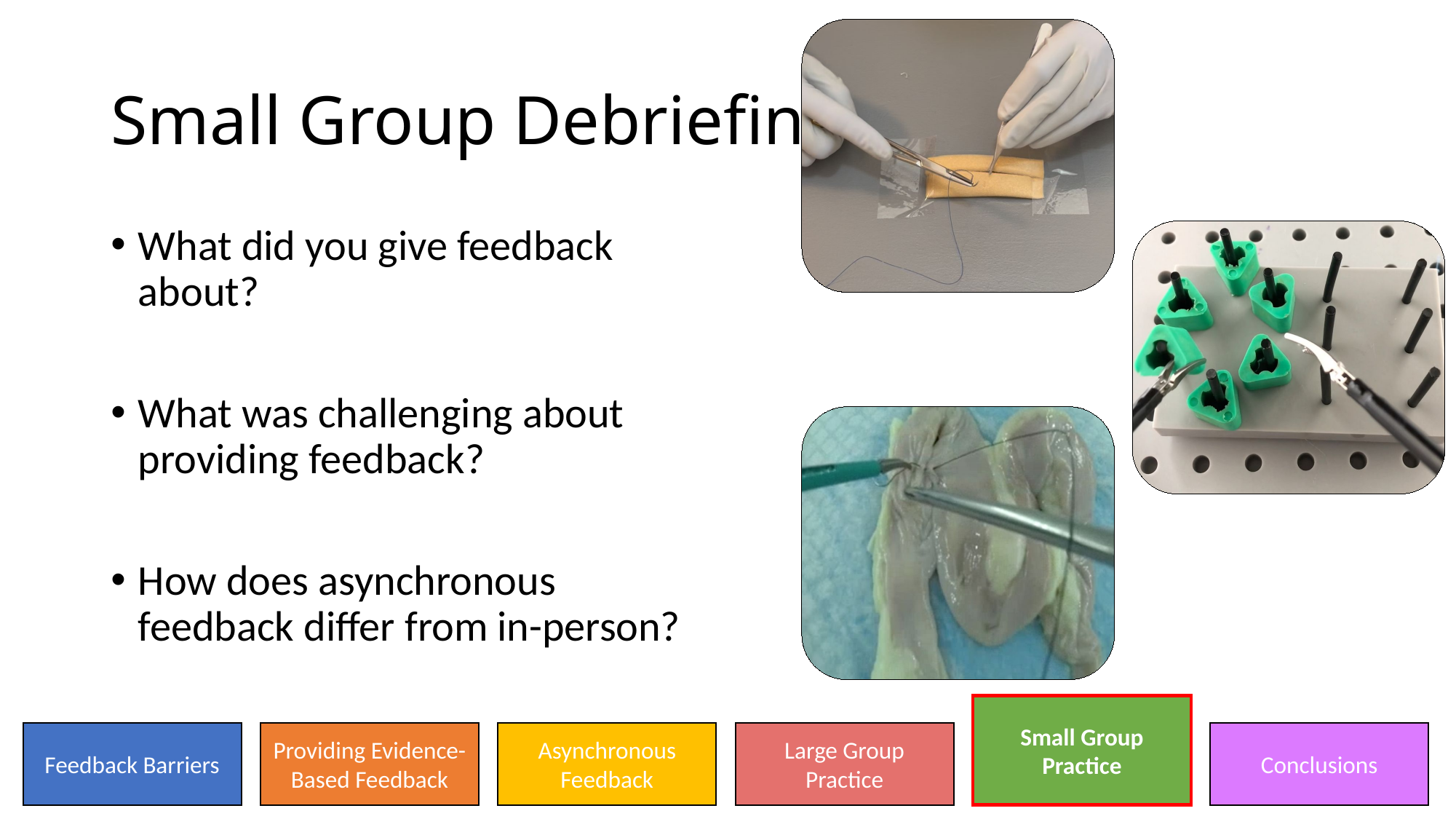

# Small Group Debriefing
What did you give feedback about?
What was challenging about providing feedback?
How does asynchronous feedback differ from in-person?
Small Group Practice
Feedback Barriers
Providing Evidence-Based Feedback
Asynchronous Feedback
Large Group Practice
Conclusions

## Slide 27
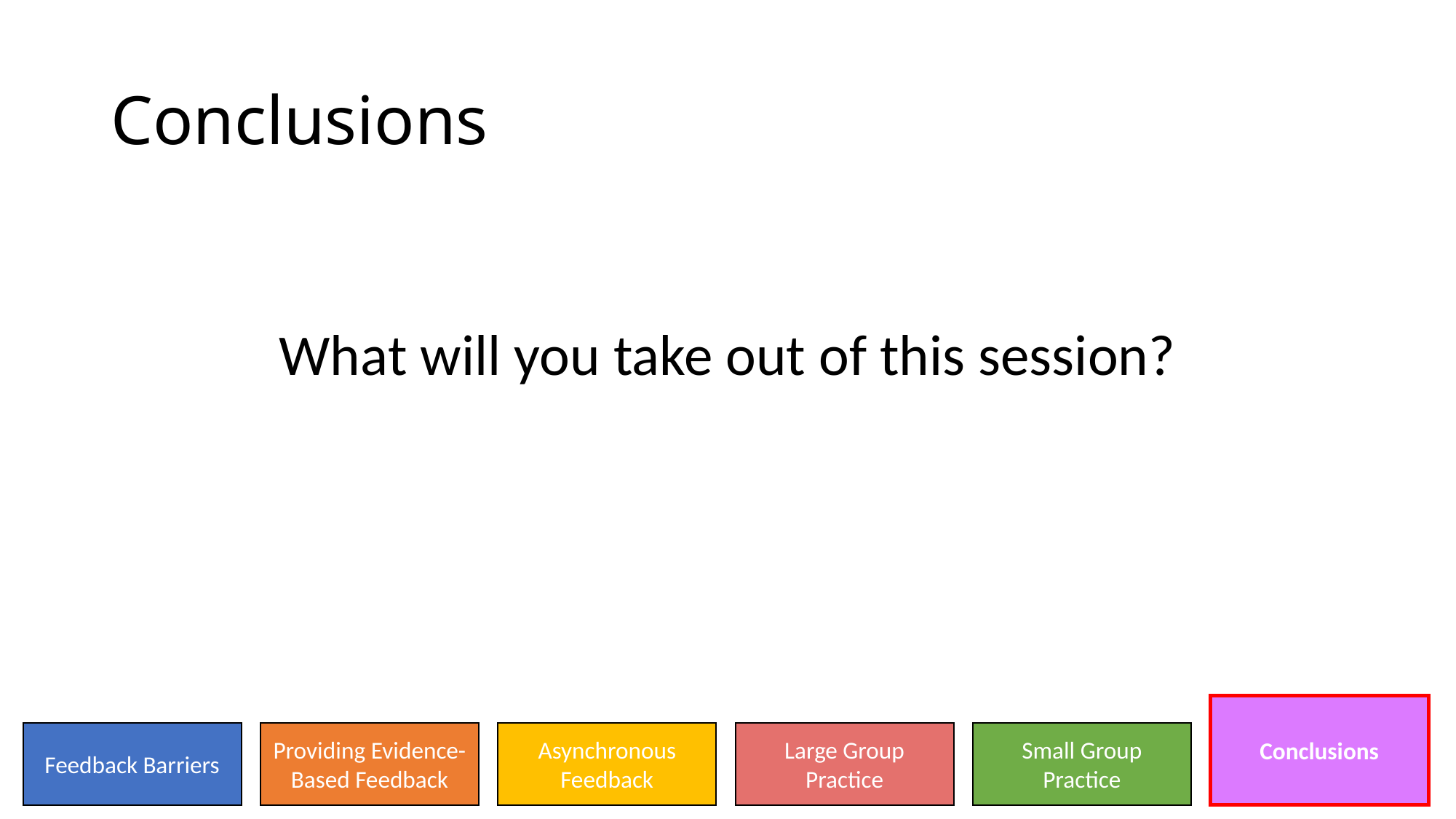

# Conclusions
What will you take out of this session?
Conclusions
Feedback Barriers
Providing Evidence-Based Feedback
Asynchronous Feedback
Large Group Practice
Small Group Practice

## Slide 28
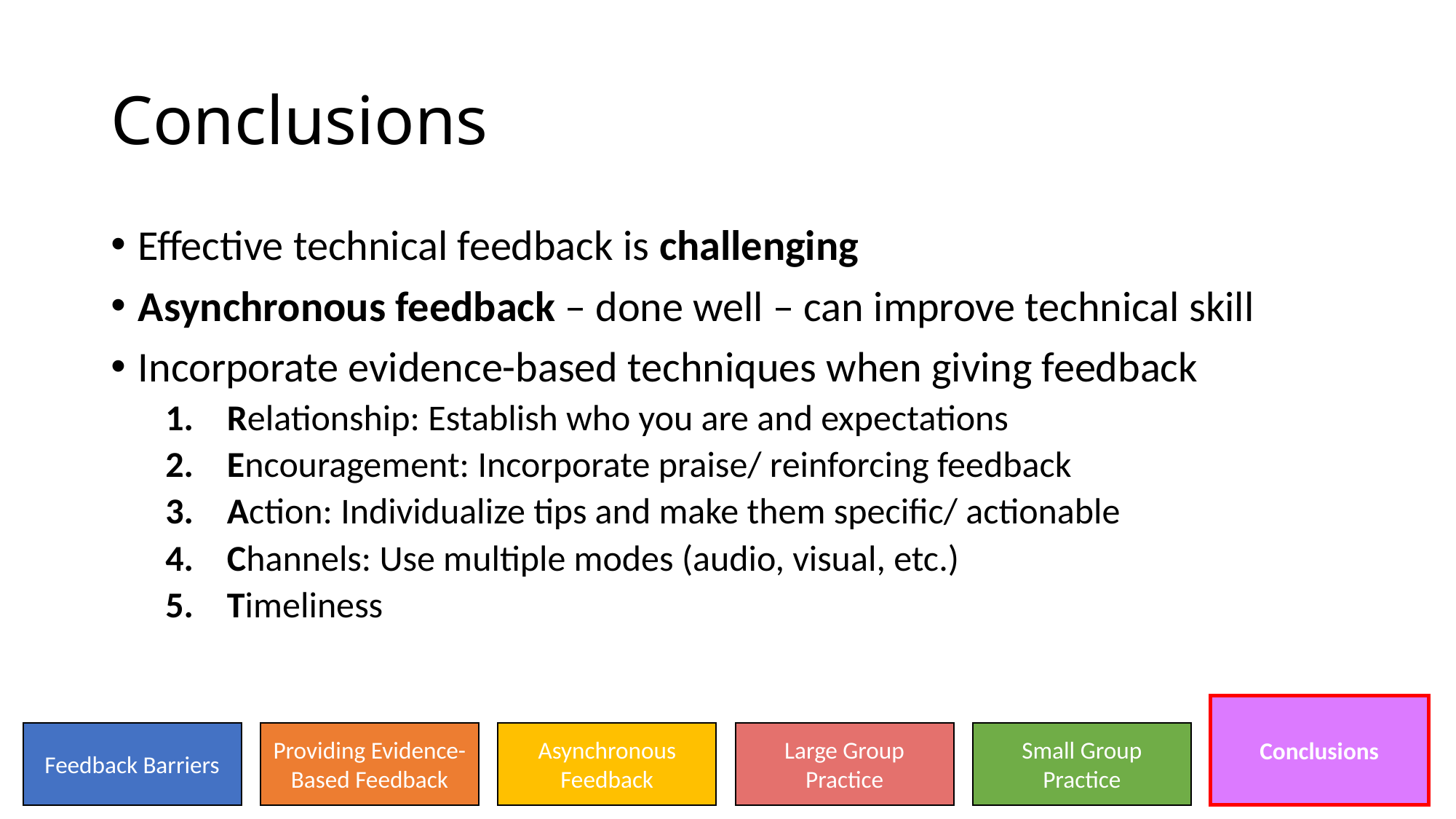

# Conclusions
Effective technical feedback is challenging
Asynchronous feedback – done well – can improve technical skill
Incorporate evidence-based techniques when giving feedback
Relationship: Establish who you are and expectations
Encouragement: Incorporate praise/ reinforcing feedback
Action: Individualize tips and make them specific/ actionable
Channels: Use multiple modes (audio, visual, etc.)
Timeliness
Conclusions
Feedback Barriers
Providing Evidence-Based Feedback
Asynchronous Feedback
Large Group Practice
Small Group Practice
