# Supplementary material for: Asynchronous Technical Feedback: A Workshop for Training Surgical Instructors
Source: MedEdPORTAL. 2025 Apr 25;21:11519. doi: 10.15766/mep_2374-8265.11519 (PMC12022122; doi:10.15766/mep_2374-8265.11519)
Supplement: Supplementary file 1 — Facilitator Guide.docxSlides.pptxSmall Group Video 1.mp4Small Group Video 2.mp4Small Group Video 3.mp4Questionnaire.docx [file mep_2374-8265.11519-s001.zip › F. Questionnaire.docx]

**Appendix F - Postworkshop Questionnaire**

*Use this appendix after the workshop to assess participants’ attitudes and knowledge.*

**First, we would like to ask you about your experience to help improve future iterations of the workshop.**

How likely are you to use the skills that you learned in this workshop?

- Extremely unlikely
- Somewhat unlikely
- Neither likely nor unlikely
- Somewhat likely
- Extremely likely

How likely are you to recommend this workshop to others?

- Extremely unlikely
- Somewhat unlikely
- Neither likely nor unlikely
- Somewhat likely
- Extremely likely

***Before*** this workshop, how confident ***were you*** in providing asynchronous technical feedback?

- Extremely unconfident
- Somewhat unconfident
- Neither confident nor unconfident
- Somewhat confident
- Extremely confident

***After*** this workshop, how confident ***are you now*** in providing asynchronous technical feedback?

- Extremely unconfident
- Somewhat unconfident
- Neither confident nor unconfident
- Somewhat confident
- Extremely confident

What are the strengths of this session?

What can be improved in this session?

**Next, we would like to ask about some of the things that you learned during the workshop.**

What are two barriers to providing *technical* feedback?

What are two situations in which you could use *asynchronous technical* feedback?

What are two ways to improve the quality of *asynchronous technical* feedback?

**Finally, we would like to ask some questions to get a sense of who has participated in the workshop.**

How much instruction have you had for providing asynchronous feedback?

- None
- Very little
- Some
- A fair amount
- A great deal

What best describes your current role?

- Medical student
- Resident
- Fellow (finished residency)
- Attending
- Other: ________________

*If medical student:*

In what year did you start medical school? _______

*If resident:*

In what year did you start residency? _______

*If fellow:*

In what year did you start fellowship? _______

*If attending:*

In what year did you finish training? _______

What is your specialty?

What other comments do you have about the session?
