# Supplementary material for: Asynchronous Technical Feedback: A Workshop for Training Surgical Instructors
Source: MedEdPORTAL. 2025 Apr 25;21:11519. doi: 10.15766/mep_2374-8265.11519 (PMC12022122; doi:10.15766/mep_2374-8265.11519)
Supplement: Supplementary file 1 — Facilitator Guide.docxSlides.pptxSmall Group Video 1.mp4Small Group Video 2.mp4Small Group Video 3.mp4Questionnaire.docx [file mep_2374-8265.11519-s001.zip › _Educational Summary Report_11519.pdf]

MedEdPORTAL® The AAMC Journal of Teaching and Learning Resources

Original Publication Open Access

Asynchronous Technical Feedback: A Workshop for Training Surgical Instructors

Riley Brian, MD, MAEd*, Camilla Gomes, MD, MSc, Úrsula Figueroa Fernández, MD, Caitlin Silvestri, MD, Sarah Lund, MD, Enrique Cruz, MD, Sergio Navarro, MD, MBA, Julián Varas, MD, MSc, Cristian Jarry, MD, MSc, Patricia S. O'Sullivan, EdD

*Corresponding author: riley.brian@ucsf.edu

Abstract

Introduction: Asynchronous learning is an efficient method for surgical trainees to gain technical skills by practicing in low-stakes and convenient settings. Effective asynchronous learning requires feedback. Prior work has highlighted the need to train surgical instructors in providing asynchronous technical feedback, as this involves unique skills related to giving feedback on learners' videos. While many existing curricula focus on optimal feedback practices, there remains a gap with regard to asynchronous technical feedback materials. **Methods:** Following Kern's six-step approach to curriculum development, we developed a 60-minute workshop, for participants across multiple contexts, on best practices for effective asynchronous technical feedback. We conducted a pilot workshop and then iteratively adjusted the flow and materials for subsequent workshop sessions. We followed survey design principles to create a postworkshop questionnaire evaluating how well the workshop addressed three of the educational objectives. **Results:** Forty-six participants attended four iterations of the workshop across three cities. Seven participants attended the pilot session of the workshop, and 39 participants joined the subsequent workshops. Of these 39 participants, 33 (85%) completed the questionnaire. Twenty-eight (85%) of 33 participants indicated that they could state a barrier to providing technical feedback after the workshop, while 30 (91%) of 33 could provide a situation in which to use asynchronous technical feedback. Twenty-seven (82%) of 33 could state a way in which to improve the quality of asynchronous technical feedback. **Discussion:** Educators may use these materials to equip instructors with tools for effectively giving learners the feedback needed for asynchronous technical skill acquisition.

Keywords
Feedback, Asynchronous Learning, Clinical/Procedural Skills Training, Surgery

Educational Objectives

By the end of this workshop, participants will be able to:

1. Identify characteristics of and barriers to providing high-quality technical feedback.
2. Describe evidence-based techniques to provide high-quality technical feedback.
3. Recognize technical skills for which asynchronous technical feedback can promote trainee practice and skill acquisition.
4. Apply evidence-based feedback techniques and produce high-quality asynchronous technical feedback.

Citation:
Brian R, Gomes C, Figueroa Fernández Ú, et al. Asynchronous technical feedback: a workshop for training surgical instructors. *MedEdPORTAL.* 2025;21:11519.
https://doi.org/10.15766/mep_2374-8265.11519

Introduction

Asynchronous learning permits surgical trainees to acquire technical skills at their own pace, when time arises between clinical duties.[1] Several published reports have described curricula and assessment evidence suggesting the promise of asynchronous technical skill development.[2-6] However, feedback remains a key component of efficient and appropriate technical skill acquisition.[7] Previous work has shown the inefficacy of technical skill practice without feedback.[8] Indeed, the theory of deliberate practice incorporates timely feedback as a key pillar of skill development. This theory posits that inadequate feedback contributes to skill stagnation.[9]

Feedback, whether for synchronous or asynchronous technical skill development, can vary substantially in quality and usefulness. Effective feedback builds on relationships, incorporates both reinforcement and correction, focuses on actionable changes, employs multimodal communication, and

Copyright © 2025 Brian et al. This is an open-access publication distributed under the terms of the Creative Commons Attribution license.

is timely.[10-13] Some of these factors change in asynchronous settings, during which instructors may provide feedback on learners' standardized patient care interactions, notes, or technical skill videos. In this asynchronous setting, feedback cannot be presented in real-time with an interactive conversation between learner and teacher. As such, there are certain considerations specific to asynchronous feedback. These considerations may include the need for an initial in-person synchronous session and practice with any involved technology.[14,15] However, several best practices for feedback translate across contexts.

Many existing curricula focus on how to provide feedback in a variety of settings.[16-18] A number of useful models to facilitate feedback conversations have been proposed in these curricula and other work.[19,20] Despite the known best practices for technical feedback and the published considerations related to the asynchronous setting, no other curricula–whether in *MedEdPORTAL* or other sources–have focused on technical feedback in asynchronous settings. As such, instructors providing asynchronous technical feedback may lack formal education on how to implement it. Our prior work specifically underscored the need for training instructors on known best practices in this area, as we observed substantial variation in asynchronous technical feedback quality.[21]

To fill the gap in training procedural and surgical instructors on asynchronous technical feedback skills, we have described the development, implementation, and evaluation of a workshop that builds from technical feedback generally to asynchronous technical feedback specifically.

Methods

Development

Following Kern's six-step approach to curriculum development, we began with problem identification and a needs assessment through a literature review and discussion with 12 surgical learners (Table 1).[22] We used these findings to create the session educational objectives. Based on the objectives, we designed an interactive session involving a mix of didactic content, participant discussion, and hands-on practice. We reviewed and iteratively refined the educational strategies and materials prior to first implementation. Given our plans to trial this workshop in multiple contexts, we developed a facilitator guide to ensure uniform implementation (Appendix A). The main session materials comprised a set of 28 slides (Appendix B) and three videos for small-group review (Appendices C, D, and E).

Implementation

We invited medical students, residents, faculty, and others with interest in asynchronous technical feedback to attend the workshop. We did not include any specific prerequisites for participants. We began the session by introducing the session objectives and asking participants key reflective questions about feedback and its challenges. We then turned to evidence-based and previously published methods to promote effective technical feedback,[10-13,16-20] framed using the REACT model that we developed to outline best practices (Appendix B). The REACT model emphasizes the importance of several considerations for technical feedback: relationships, encouragement, action, channels/multimodality, and timeliness. After discussing these evidence-based strategies for providing technical feedback, we focused on asynchronous technical feedback. We highlighted the affordances and constraints of asynchronous technical feedback and emphasized how the previously discussed strategies apply to the asynchronous setting. We also had a group discussion about the logistics of using asynchronous feedback. After this discussion, we conducted a large-group practice, in which participants watched a video of a student performing a technical skill and then discussed feedback strategies together. Following the large-group practice, we divided participants into smaller groups for guided independent practice. Small groups reviewed a set of shared videos and practiced providing feedback using an online platform. Finally, we led a debrief about the small-group practice session and emphasized key session takeaways.

Serial Iteration

We first piloted the curriculum with a group of seven participants (representing faculty, residents, and medical students). We chose to start with a small pilot to identify major areas for workshop changes. At the end of the pilot, we discussed the session objectives, flow, and materials with participants. We made several changes to the workshop at this point, including adjusting the materials to better align with the objectives and changing the order of the session to better enable participant understanding. We conducted a second, third, and fourth session of the workshop following the initial pilot. Participants from these sessions had fewer suggestions for workshop improvement, though we did make some adjustments to the example videos and slides to improve clarity. We also trialed the workshop in 45-minute, 60-minute, and 90-minute versions, based on varied opinions about workshop length from participants.

Evaluation and Analysis

We created a postworkshop questionnaire for participants to evaluate the first three workshop educational objectives.

Copyright © 2025 Brian et al. This is an open-access publication distributed under the terms of the Creative Commons Attribution license.

Table 1. Curriculum Development Based on Kern's Six-Step Approach

Step	Results
Problem identification	• There is substantial variation in the quality of asynchronous technical feedback given to learners during technical skill development<br>• Current instructors do not frequently receive instruction on how to provide asynchronous technical feedback<br>• There is a gap in curricula equipping instructors with tools to provide such feedback
Targeted needs assessment	• Several key aspects of feedback delivery represent areas for instructor development
Goals and objectives	• Session participants will (1) identify characteristics of and barriers to providing high-quality technical feedback, (2) describe evidence-based techniques to provide high-quality technical feedback, (3) recognize technical skills for which asynchronous technical feedback can promote trainee practice and skill acquisition, and (4) apply evidence-based feedback techniques and produce high-quality asynchronous technical feedback
Educational strategies	• Didactic introduction<br>• Group discussion<br>• Hands-on practice
Implementation	• Formal overview of feedback challenges, evidence-based feedback techniques, and asynchronous feedback<br>• Large-group practice of giving high-quality asynchronous technical feedback<br>• Small-group practice of giving high-quality asynchronous technical feedback<br>• Debriefing
Evaluation	• Real-time feedback from participants<br>• Questionnaire to evaluate educational objectives 1, 2, and 3

Following the survey design process outlined by Artino, La Rochelle, Dezee, and Gehlbach,[23] we first performed a literature review, and then conducted a focus group with 14 surgical education research fellows to understand and define the constructs in our questionnaire. We synthesized prior published questionnaires with findings from our focus group to develop items relevant to the workshop.[24-26] We discussed items with experts among the author group, including experts in survey design and experts in asynchronous technical feedback. We piloted the questionnaire during the pilot session of the curriculum and refined the items. We did not retain responses from this pilot session because we made subsequent changes to the questionnaire. We distributed the final questionnaire to all participants in the second, third, and fourth iterations of the curriculum. This questionnaire contained three sections and 14 items focusing on attitudes, retrospective and postworkshop confidence, knowledge around technical and asynchronous technical feedback, and participant information (Appendix F).

We generated descriptive statistics from the results of the questionnaire and used a Wilcoxon signed-rank test to compare paired ordinal data. We used a Wilcoxon rank-sum test to compare medical student and non–medical student participant groups. For statistical analysis, we used Stata/IC 16.1 software for Mac (College Station, TX).

Context

We implemented this workshop in 2024 for educational and surgical audiences across multiple sites. Facilitators for this workshop all had some prior experience providing technical feedback, though they had varied experience with asynchronous technical feedback. While preparing for the workshop, all facilitators worked together to further practice and refine their skills with asynchronous technical feedback.

We conducted the workshop in classrooms with projectors at sites in three cities: San Francisco, California (United States), Santiago (Chile), and Orlando, Florida (United States). For small-group activities, we asked participants to bring computers with internet access capability. During these sessions, we used the free online platform C1Do1 to allow participants to practice providing asynchronous technical feedback. Only the session leader needed to create an account to use this platform for the workshop. Other online platforms (e.g., Practice XYZ) have similar functionality. Regardless of the specific platform used, a program to facilitate multimodal feedback helped with hands-on practice. We hosted two of the workshop iterations as offerings at conferences and two of the workshop iterations as independent events.

The University of California, San Francisco Institutional Review Board exempted this curricular development and evaluation from review (UCSF IRB no. 24-41011, 2024).

Results

Forty-six participants attended four iterations of the workshop across three cities. Seven participants attended the first pilot session of the workshop, and 39 participants joined the subsequent workshops. Of these 39 participants, 33 (85%) completed all or part of the questionnaire and were included in the final analysis. Participants represented a range of roles, including Medical students ($n = 14$), Residents ($n = 10$), attending ($n = 7$), and Other ($n = 2$). Participants who reported a specialty ($n = 15$) worked most commonly in general surgery ($n = 11$).

Copyright © 2025 Brian et al. This is an open-access publication distributed under the terms of the Creative Commons Attribution license.

The questionnaire first assessed confidence and attitudes about the workshop. Though many participants ($n = 17$, 52%) reported having received no or very little prior instruction in providing asynchronous technical feedback, most participants ($n = 18$, 55%) stated that they were confident in providing asynchronous technical feedback before the workshop. The preworkshop confidence level, rated on a scale of 1–5 (1 = *extremely unconfident*, 2 = *somewhat unconfident*, 3 = *neither confident nor unconfident*, 4 = *somewhat confident*, 5 = *extremely confident*), was higher among medical student participants (median preworkshop confidence 4) than among all nonmedical student participants (median preworkshop confidence 3; $p < .001$). Seventeen (52%) of 33 participants reported that their confidence in providing asynchronous technical feedback increased following the workshop ($p < .001$; Figure 1). Of the seven participants who reported feeling unconfident in providing asynchronous technical feedback before the workshop, all seven reported an increase in confidence by the end of the workshop. Medical student and non–medical student participants reported similar levels of postworkshop confidence (median postworkshop confidence 4 in both groups; $p = .54$). Twenty-four (73%) of 33 participants reported that they would recommend the workshop to others, and 25 (76%) of 33 reported that they were likely to use the skills they learned in the workshop.

The questionnaire next assessed our educational objectives by determining participants' postworkshop knowledge of feedback barriers and the use of asynchronous technical feedback (Figure 2). Twenty-eight (85%) of 33 participants were able to state a barrier to providing technical feedback after the workshop, while 30 (91%) of 33 were able to provide a situation in which to use asynchronous technical feedback. Twenty-seven (82%) of 33 were able to state a way in which to improve the quality of asynchronous technical feedback.

In narrative comments (summarized in Table 2), participants reflected on several strengths of the workshop, including the easy-to-use REACT mnemonic for improving asynchronous technical feedback quality, the clarity of the presented information, and the hands-on nature of the session. Participants also suggested areas for improvement. The most common suggestion—proposed by 11 respondents in free-text responses—was related to either insufficient or excess time in the workshop. Most of those who commented on workshop duration participated in the 45-minute version of the session and felt it to be too short. Others suggested that the small-group practice be shifted to individual practice to give every participant the chance to provide asynchronous technical feedback.

Discussion

In this report, we have described a newly developed workshop to teach procedural and surgical instructors how to provide effective asynchronous technical feedback. The workshop can be easily implemented with the use of few materials and fills a gap in available materials to train instructors in an increasingly important skill. Most participants reported increased confidence in providing asynchronous technical feedback after attending the workshop. Furthermore, most participants were able to display

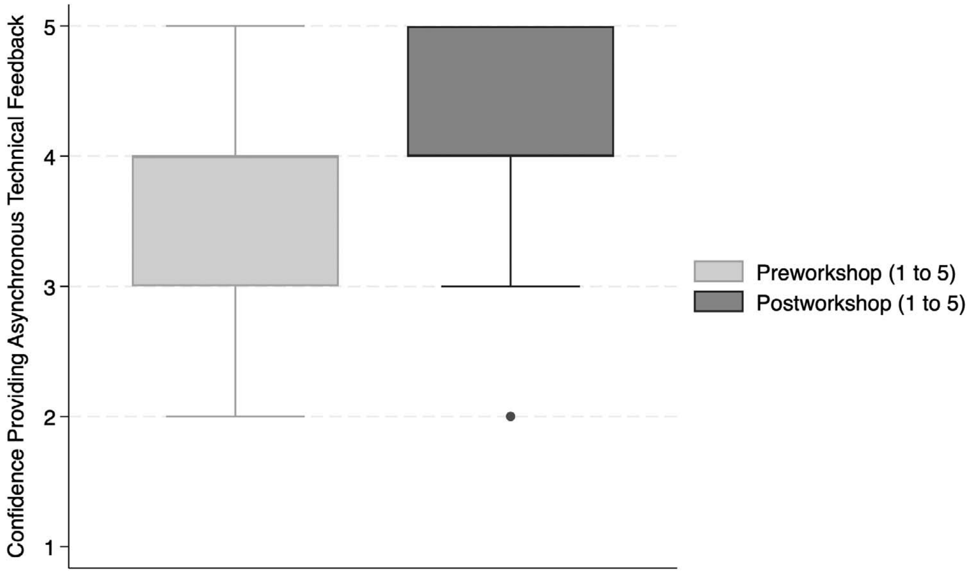

Figure 1. On the questionnaire (see Appendix F), participants reported that they had increased confidence in providing asynchronous technical feedback after workshop participation compared to before workshop participation ($p < .001$). Box plots represent the median (interquartile range) confidence rating on a scale of 1 to 5.

Copyright © 2025 Brian et al. This is an open-access publication distributed under the terms of the Creative Commons Attribution license.

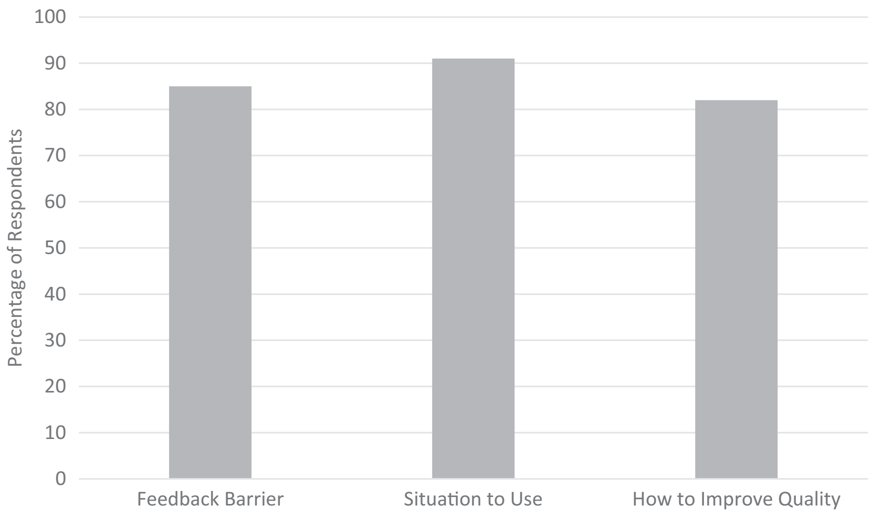

Figure 2. Responses to the questionnaire (see Appendix F) indicated that, after the workshop, most participants were able to state a barrier to providing asynchronous technical feedback, were able to provide a situation in which to use asynchronous technical feedback, and were able to state a way in which to improve the quality of asynchronous technical feedback.

relevant knowledge related to key aspects of the first three workshop educational objectives.

This workshop adds to the existing literature promoting feedback skills in medical education. Prior studies have outlined easy-to-use models for instructors to facilitate effective feedback, often for giving feedback in synchronous settings.[16-20] Other work has emphasized the efficacy of programs training novices—such as some of those who participated in our workshop—to provide technical feedback.[27] Our report contributes to this literature through its specific focus on providing asynchronous technical feedback, an increasingly important type of feedback given changes in training structures and the importance of equipping trainers with effective and diverse feedback strategies. Asynchronous technical feedback practice represents a promising way for trainees to attain skills in a low-stakes setting and at convenient locations and times for both trainees and faculty.[28,29]

Table 2. Summary of Narrative Comments From Workshop Participants

Theme	Participant Comments
Strengths	Introduction and general overview
	Clear objectives
	Summary of evidence for feedback practices
	Examples
	REACT mnemonic
	Hands-on and interactive components
	Clear presentation
Weaknesses	Workshop duration
	Heterogeneity of small-group participants
	Lack of nonsurgical examples
	Repetitive nature of session
	Understanding how to use the technology

Abbreviation: REACT, relationship, encouragement, action, channels, timeliness.

As part of the evaluation and feedback step of Kern's approach to curriculum development, we adjusted the workshop across its four iterations.[22] Following the initial pilot, we adapted the workshop in accordance with participants' suggestions regarding materials and flow. Many participants had (occasionally conflicting) suggestions regarding workshop duration. After collating all responses and running the workshop in 45-, 60-, and 90-minute versions, we identified the 60-minute version as providing an appropriate balance.

There are a number of limitations and lessons learned. First, we measured knowledge-related outcomes for our first three educational objectives through a postworkshop questionnaire. We did not measure changes in participants' behavior or results. Also, we did not directly measure the fourth objective. Participants practiced applying evidence-based feedback techniques to sample trainee videos during the workshop, though we did not assess their work or measure retention. Furthermore, we implemented this workshop in academic centers at three sites. The workshop is most likely to be relevant to similar academic settings. Additionally, we did not require participants to have prior background with technical feedback. As such, there was a wide range in participants' roles and experiences, with more than one-third of participants being medical students. This created the challenge of balancing basic and advanced topics tailored to the specific participants. We

Copyright © 2025 Brian et al. This is an open-access publication distributed under the terms of the Creative Commons Attribution license.

found this topic to be relevant to many types of participants, particularly given the increasing use of peer feedback in synchronous and asynchronous technical feedback. Peer feedback allows both the feedback giver and recipient, even when inexperienced, to benefit from the feedback process.[21,30] Nonetheless, use of suggested prerequisites to better frame the workshop for potential participants may address this problem. Alternatively, hosting the workshop in smaller sessions but with more homogenous groups may allow for a more focused level of discourse. For example, participants with limited technical experience (e.g., medical students) may be better able to review basic technical skill videos. Based on the group of participants, those implementing this workshop may choose to focus on just one of the small-group videos (Appendices C, D, and E). Finally, some participants had mixed responses with regard to recommending the workshop and changes in confidence following the workshop. Of note, many participants started the workshop with a high level of confidence, which may have limited any subsequent increase in confidence level postworkshop. Medical students reported higher preworkshop confidence levels on average. Confidence as an outcome measure has multiple limitations, as an individual may be confident but lacking in skill. This may have additionally been limited given that the questions regarding participants' confidence levels pre- and postworkshop were both asked after the session. Future work could evaluate the efficacy of feedback given by session attendees.

Next directions include incorporating the workshop into annual training for procedural and surgical instructors who provide asynchronous technical feedback. Overall, as asynchronous technical feedback practice becomes increasingly prevalent, equipping procedural and surgical instructors with the skills to provide feedback in this setting will be essential.

Appendices

A. Facilitator Guide.docx

B. Slides.pptx

C. Small Group Video 1.mp4

D. Small Group Video 2.mp4

E. Small Group Video 3.mp4

F. Questionnaire.docx

All appendices are peer reviewed as integral parts of the Original Publication.

Riley Brian, MD, MAEd: Fourth-Year Resident, Department of Surgery, University of California, San Francisco; ORCID: https://orcid.org/0000-0002-7462-3214

Camilla Gomes, MD, MSc: Research Resident, Department of Surgery, University of California, San Francisco

Úrsula Figueroa Fernández, MD: Second-Year Resident, Department of Surgery, Universidad de Desarrollo

Caitlin Silvestri, MD: Research Resident, Department of Surgery, New York Presbyterian/Columbia University Irving Medical Center

Sarah Lund, MD: Fifth-Year Resident, Department of Surgery, Mayo Clinic

Enrique Cruz, MD: Research Fellow, Center for Simulation and Experimental Surgery, Faculty of Medicine, UC-Christus Health Network, Pontificia Universidad Católica de Chile

Sergio Navarro, MD, MBA: Research Fellow, Department of Surgery, Mayo Clinic

Julián Varas, MD, MSc: Associate Professor, Division of Surgery, Faculty of Medicine, UC-Christus Health Network, Pontificia Universidad Católica de Chile

Cristian Jarry, MD, MSc: Research Associate, Division of Surgery, Faculty of Medicine, UC-Christus Health Network, Pontificia Universidad Católica de Chile

Patricia S. O'Sullivan, EdD: Professor of Medicine and Surgery, University of California, San Francisco, School of Medicine

Disclosures

Dr. Julian Varas is the Founder of Training Competence, an official spinoff startup from the Pontificia Universidad Católica de Chile. Training Competence and the Pontificia Universidad Católica de Chile are the proprietors of the rights and distribution of the C1DO1 platform used in this workshop. We do not mention this platform by name in the publication.

Funding/Support

None to report.

Ethical Approval

The UCSF Institutional Review Board reviewed this project.

References

1. Coronel BV, Álvarez RIS, Riveros FB, et al. Minor surgery course using remote and asynchronous feedback: training wherever, whenever. *Global Surg Educ*. 2023;2:109. https://doi.org/10.1007/s44186-023-00175-9
2. Ulloa G, Neyem A, Escalona G, Ortiz C, Varas J. Remote asynchronous feedback for unsupervised laparoscopic training: the "LAPP" platform. *Arq Bras Cir Dig*. 2023;35:e1712. https://doi.org/10.1590/0102-672020220002e1712

Copyright © 2025 Brian et al. This is an open-access publication distributed under the terms of the Creative Commons Attribution license.

3. Gaete MI, Belmar F, Cortés M, et al. Remote and asynchronous training network: from a SAGES grant to an eight-country remote laparoscopic simulation training program. *Surg Endosc*. 2023; 37(2):1458-1465. https://doi.org/10.1007/s00464-022-09386-5

4. Brian R, Bayne D, Ito T, et al. An at-home laparoscopic curriculum for junior residents in surgery, obstetrics-gynecology, and urology. *MedEdPORTAL*. 2024;20:11405. https://doi.org/10.15766/mep_2374-8265.11405

5. Wile RK, Brian R, Rodriguez N, Chern H, Cruff J, O'Sullivan PS. Home practice for robotic surgery: a randomized controlled trial of a low-cost simulation model. *J Robot Surg*. 2023;17(5): 2527-2536. https://doi.org/10.1007/s11701-023-01688-7

6. Rammell J, Matthan J, Gray M, et al. Asynchronous unsupervised video-enhanced feedback as effective as direct expert feedback in the long-term retention of practical clinical skills: randomised trial comparing 2 feedback methods in a cohort of novice medical students. *J Surg Educ*. 2018;75(6):1463-1470. https://doi.org/10.1016/j.jsurg.2018.03.013

7. Yilmaz R, Fazlollahi AM, Winkler-Schwartz A, et al. Effect of feedback modality on simulated surgical skills learning using automated educational systems- a four-arm randomized control trial. *J Surg Educ*. 2024;81(2):275-287. https://doi.org/10.1016/j.jsurg.2023.11.001

8. Tejos R, Avila R, Inzunza M, et al. Impact of a simulated laparoscopic training program in a three-year general surgery residency. *Arq Bras Cir Dig*. 2019;32(2):e1436. https://doi.org/10.1590/0102-672020190001e1436

9. Ericsson KA. Deliberate practice and acquisition of expert performance: a general overview. *Acad Emerg Med*. 2008; 15(11):988-994. https://doi.org/10.1111/j.1553-2712.2008.00227.x

10. Collings AT, Doster DL, Longtin K, Choi J, Torbeck L, Stefanidis D. Surgical resident perspectives on the preferred qualities of effective intraoperative teachers: a qualitative analysis. *Acad Med*. 2023;98(5):629-635. https://doi.org/10.1097/ACM.0000000000005131

11. Wong EY, Chu TN, Ma R, et al. Development of a classification system for live surgical feedback. *JAMA Netw Open*. 2023;6(6): e2320702. https://doi.org/10.1001/jamanetworkopen.2023.20702

12. Vygotsky LS, Cole M, Jolm-Steiner V, Scribner S, Souberman E, eds. *Mind in Society: Development of Higher Psychological Processes*. Harvard University Press; 1980. https://doi.org/10.2307/j.ctvjf9vz4

13. Neal CJ, Durning SJ, Dharmapurikar R, McDaniel KE, Lad SP, Haglund MM. From their eyes: what constitutes quality formative written feedback for neurosurgery residents. *J Surg Educ*. 2023; 80(3):323-330. https://doi.org/10.1016/j.jsurg.2022.10.003

14. Kimura R, Matsunaga M, Barroga E, Hayashi N. Asynchronous e-learning with technology-enabled and enhanced training for continuing education of nurses: a scoping review. *BMC Med Educ*. 2023;23(1):505. https://doi.org/10.1186/s12909-023-04477-w

15. Mears K, Blake L. Nursing librarians cultivating evidence-based practice through an asynchronous online course. *J Contin Educ Nurs*. 2017;48(9):420-424. https://doi.org/10.3928/00220124-20170816-08

16. Sargeant J, Armson H, Driessen E, et al. Evidence-informed facilitated feedback: the R2C2 feedback model. *MedEdPORTAL*. 2016;12:10387. https://doi.org/10.15766/mep_2374-8265.10387

17. Aagaard E, Czernik Z, Rossi C, Guiton G. Giving effective feedback: a faculty development online module and workshop. *MedEdPORTAL*. 2010;6:8119. https://doi.org/10.15766/mep_2374-8265.8119

18. Pettit J. Workshop on giving, receiving, and soliciting feedback. *MedEdPORTAL*. 2011;7:9060. https://doi.org/10.15766/mep_2374-8265.9060

19. Fainstad T, McClintock AA, Van der Ridder MJ, Johnston SS, Patton KK. Feedback can be less stressful: medical trainee perceptions of using the prepare to ADAPT (Ask-Discuss-Ask-Plan Together) framework. *Cureus*. 2018;10(12):e3718. https://doi.org/10.7759/cureus.3718

20. Hoon A, Oliver E, Szpakowska K, Newton P. Use of the 'Stop, Start, Continue' method is associated with the production of constructive qualitative feedback by students in higher education. *Assess Eval Higher Educ*. 2014;40(5):755-767. https://doi.org/10.1080/02602938.2014.956282

21. Brian R, Cowan B, Knox JA, et al. Comparing peer and faculty feedback for asynchronous laparoscopic skill acquisition. *J Surg Educ.* 2024;81(8):1154-1160. https://doi.org/10.1016/j.jsurg.2024.05.011

22. Thomas P, Kern D, Hughes M, Chen B. *Curriculum Development for Medical Education: A Six-Step Approach*. 3rd ed. Johns Hopkins Press; 2016.

23. Artino AR, La Rochelle JS, Dezee KJ, Gehlbach H. Developing questionnaires for educational research: AMEE Guide No. 87. *Med Teach*. 2014;36(6):463-474. https://doi.org/10.3109/0142159X.2014.889814

24. Anderson MJ, Ofshteyn A, Miller M, Ammori J, Steinhagen E. "Residents as Teachers" workshop improves knowledge, confidence, and feedback skills for general surgery residents. *J Surg Educ*. 2020;77(4):757-764. https://doi.org/10.1016/j.jsurg.2020.01.010

25. Yau BN, Chen AS, Ownby AR, Hsieh P, Ford CD. Soliciting feedback on the wards: a peer-to-peer workshop. *Clin Teach*. 2020;17(3):280-285. https://doi.org/10.1111/tct.13069

26. Miller S, Shipper E, Hasty B, et al. Introductory surgical skills course: technical training and preparation for the surgical environment. *MedEdPORTAL*. 2018;14:10775. https://doi.org/10.15766/mep_2374-8265.10775

Copyright © 2025 Brian et al. This is an open-access publication distributed under the terms of the Creative Commons Attribution license.

27. Espinoza VD, Jaras IM, Schilling MM, et al. Train the trainers: a comparative analysis of medical students and residents as trainers for undergraduate med-school learners in surgical skills. *Global Surg Educ*. 2023;2:110. https://doi.org/10.1007/s44186-023-00193-7

28. Park KM, Rashidian N, Anderson C, et al. Evidence-based guidelines on the use of virtual surgical education pertaining to the domains of cognition and curriculum, psychomotor skills training, and faculty development and mentorship. *Ann Surg*. 2022;276(1):e6-e15. https://doi.org/10.1097/SLA.0000000000005014

29. Blackhall VI, Cleland J, Wilson P, Moug SJ, Walker KG. Barriers and facilitators to deliberate practice using take-home laparoscopic simulators. *Surg Endosc*. 2019;33(9):2951-2959. https://doi.org/10.1007/s00464-018-6599-9

30. Vaughn CJ, Kim E, O'Sullivan P, et al. Peer video review and feedback improve performance in basic surgical skills. *Am J Surg*. 2016;211(2):355-360. https://doi.org/10.1016/j.amjsurg.2015.08.034

Received: June 13, 2024
Accepted: March 16, 2025
Published: April 25, 2025

Copyright © 2025 Brian et al. This is an open-access publication distributed under the terms of the Creative Commons Attribution license.
